# Sensitivity of endogenous autofluorescence in HeLa cells to the application of external magnetic fields

**DOI:** 10.1038/s41598-023-38015-x

**Published:** 2023-07-04

**Authors:** Mariia Uzhytchak, Barbora Smolková, Adam Frtús, Alexandr Stupakov, Mariia Lunova, Federica Scollo, Martin Hof, Piotr Jurkiewicz, Gareth John Sullivan, Alexandr Dejneka, Oleg Lunov

**Affiliations:** 1 Department of Optical and Biophysical Systems, Institute of Physics of the Czech Academy of Sciences, Prague, 18221 Czech Republic; 2 Institute for Clinical and Experimental Medicine (IKEM), Prague, 14021 Czech Republic; 3 J. Heyrovský Institute of Physical Chemistry of the Czech Academy of Sciences, Prague, 18223 Czech Republic; 4 Department of Molecular Medicine, Institute of Basic Medical Sciences, University of Oslo, Oslo, Norway; 5 Department of Pediatric Research, Oslo University Hospital, Oslo, Norway; 6 Department of Immunology, Institute of Clinical Medicine, University of Oslo, Oslo, Norway

**Keywords:** Cellular imaging, Biological fluorescence

## Abstract

Dramatically increased levels of electromagnetic radiation in the environment have raised concerns over the potential health hazards of electromagnetic fields. Various biological effects of magnetic fields have been proposed. Despite decades of intensive research, the molecular mechanisms procuring cellular responses remain largely unknown. The current literature is conflicting with regards to evidence that magnetic fields affect functionality directly at the cellular level. Therefore, a search for potential direct cellular effects of magnetic fields represents a cornerstone that may propose an explanation for potential health hazards associated with magnetic fields. It has been proposed that autofluorescence of HeLa cells is magnetic field sensitive, relying on single-cell imaging kinetic measurements. Here, we investigate the magnetic field sensitivity of an endogenous autofluorescence in HeLa cells. Under the experimental conditions used, magnetic field sensitivity of an endogenous autofluorescence was not observed in HeLa cells. We present a number of arguments indicating why this is the case in the analysis of magnetic field effects based on the imaging of cellular autofluorescence decay. Our work indicates that new methods are required to elucidate the effects of magnetic fields at the cellular level.

## Introduction

Despite decades of research, the biological effects of magnetic fields still remain a highly debatable topic without consensus over major outcomes^1–4^. Thus far, dozens of studies have ascribed a variety of biological effects to both electromagnetic as well as static magnetic fields^5–8^. It has been widely acknowledged that many investigations which deal with the biological impact of magnetic fields are hampered by shortcomings in experimental design and often also by the lack of reproducibility^1–4,6,7,9,10^. Specifically, direct attempts to replicate key findings on biological effects of magnetic fields have been largely unsuccessful^11–15^. Therefore, the jury is still out with regards to the molecular and/or biophysical foundations for these proposed cellular effects. Considering all the above-mentioned queries, studies focusing on biological effects of magnetic fields are intriguing, challenging, and timely.

Furthermore, some epidemiological studies presumed a low interrelation between residential proximity to high-voltage power lines and childhood leukemia^16–18^. Recent literature analysis showed that the majority of large-scale epidemiological studies do not support this association (for review see^19^ and references therein). In this flow of data the International Agency for Research on Cancer (IARC) classified magnetic fields of extremely low-frequencies as “possibly carcinogenic to humans” (Group 2B) in 2002, admitting that the evidence is limited^20^. Recent systematic reviews on bioeffects of weak and intermediate, static, and different frequency range, magnetic and electromagnetic fields found, that current evidence does not allow one to draw a firm conclusion for biological and health-related consequences of exposure to those fields^4,21,22^.

Numerous studies on biological effects of magnetic fields have produced many hypothesizes, proposing distinct potential mechanisms of magnetic field action on biological matter^1–3,6–8^. One hypothesis explaining biological effects of magnetic fields is alteration of biochemical reactions via so-called radical pair (RP) mechanism (RPM)^3,23,24^. Indeed, attempts to independently reproduce suggested magnetic field effects (MFEs) via RPM in different biochemical systems faced challenges^11,25–29^. Some studies have doubted the relevance of RPM hypothesis in biological systems and questioned the interpretation of results^2,30,31^. It was proposed that flavin adenine dinucleotide (FAD)-containing photoreceptors (cryptochromes) might be a key player responsible for RPM-based MFEs^32–34^. However, others showed that reactions catalyzed by flavin-dependent enzymes are unlikely to be influenced by magnetic fields^35^. Of note, external magnetic fields have shown the ability to change chemical reactions in artificial cell-free systems in vitro^3,36^. Magnetic fields may affect radical pair reactions in cell-free systems of flavin/tryptophan molecules mixtures^34,37–41^. However, cellular heterogeneity and complexity greatly affect biochemical reactions making them differ from those in test tubes^42–45^. Thus, it is important to verify results obtained from cell-free systems utilizing living cells. So far, studies evaluated the hypothesis of flavin-based RPM in cell-free systems^34,35,37^. Recently it was shown that there might be magnetic field sensitivity of FAD-based RPM at the cellular level^46^. In light of all discussed above, MFEs at the cellular level represent an intriguing and interesting finding. Understanding the spatiotemporal mechanisms of the magnetic field-induced effects will enable the deliberate exploitation of such signals in different biomedical applications. Robust, and direct demonstration of magnetic field responses at the individual cell level requires reevaluation studies that may create an important fundamental background for future studies of magnetic field effects. Here we report MFE on the autofluorescence of HeLa cells, introducing several critical control points.

## Materials and methods

### Cell culture

HeLa cells were obtained from the American Type Culture Collection (ATCC CCL-2). Cells were cultured in Minimum Essential Medium Eagle (BioConcept Ltd., Switzerland) supplemented with 10% fetal bovine serum (FBS, Thermo Fisher Scientific, US), 1% l-Glutamine 100 ×, 200 mM (Serana Europe GmbH, Germany) and 1% Penicillin/Streptomycin (Thermo Fisher Scientific, US). Cell cultures were cultivated in a humidified 5% CO_2_ atmosphere at 37 °C. Cell culture medium was replaced once a week. Cells were regularly checked for common culture contamination, such as Mycoplasma using MycoAlert Detection Assay (Lonza, Switzerland). HeLa cell line was authenticated by short tandem repeat (STR) DNA profiling (ATCC, Manassas, VA, USA).

### Magnetic field exposure setup

Magnetic field was generated by a pancake/bobbin coil of 3 cm width and 11/14 cm inner/outer diameters having 550 turns of copper wire of 0.8-mm diameter. The coil was calibrated using a laboratory Gaussmeter FW Bell 7030 and their transverse Hall probe STF71-0404-05-T with temperature compensation. The coil was adjusted directly on the top of Olympus IX3-SVR mechanical stage at a distance of 5 mm above the sample slide in order to generate appropriate field strengths of the magnetic field. Cells were placed directly inside the coil, in the central axial area on a level of the coil edge. Specific number of the coil in this area was estimated as ~ 5 mT/A. Triangular voltage waveforms with frequencies of 0.1–0.2 Hz were supplied by a standard arbitrary waveform generator OWON AG1022 (Owon, China). The driving signal was power amplified in a voltage control mode by an MP39 module mounted on an EK59 evaluation kit (APEX Microtechnology, US). During the measurements, the magnetic field was controlled by the same Gaussmeter, and transverse Hall probe positioned at the opposite upper coil edge and inside the coil, in the central axial area where cells were placed. The amplitude of the magnetic field was set to 10 or 20 mT, inside the coil, in the central axial area where cells were placed. The intensity and waveform of resultant magnetic field were identical to the original study^46^. Driving current ~ 4 A (33 V) was applied to generate 20 mT magnetic field. DC offset of the magnetic field measured without driving current does not exceed 0.1 mT and is mostly determined by the Earth’s magnetic field.

In order to treat cells with static magnetic field, we applied cylindrical (radius 5 mm; length 50 mm; residual magnetic flux density 1.4 T) bulk NdFeB magnet. Magnet was applied on the top of the 6-channels Ibidi μ-slides (Ibidi, Germany) at the central part of the channel (Fig. S1). Magnetic flux density (*B*(*x*)) at the level of cells was estimated to be ~ 500 mT (Fig. S1).

In order to cross-check that the noise from imaging system and other equipment in the laboratory does not have significant impact, we measured the background electromagnetic noise. To estimate this the voltage induced in the same bobbin coil was measured by a 16-bit acquisition board NI PCIe-6351 at 2 MSa/s sampling rate. The measured noise voltage of ~ 8 mV amplitude and 2.5 mV rms value is independent of the microscope activity (whether it is switched on/off) and fully determined by background electromagnetic noises in the laboratory (Fig. S2A). RMS value of the highest noise harmonic at 639 kHz is ~ 1.5 mV; the highest harmonic in a low-frequency range is of ~ 0.2 mV rms at power line frequency 50 Hz (Fig. S2B). Such a 50-Hz harmonic gives a leap of the magnetic field ~ 0.15 µT, which is a common level of the background magnetic noise.

It is worth noting that the applied magnetic field may be distorted due to presence of microscope structure, e.g., objectives^47,48^. The highest distortions of the applied magnetic field were observed in objectives containing large amount of ferromagnetic elements and/or when objectives were placed in a close proximity to the coil, e.g., closer than 5 mm^47^. That is why in our experiments coil was mounted at a distance of 5 mm above the sample slide. In fact, old objectives manufactured before 1960 containing significant amount of ferromagnetic elements showed large distortions of the applied magnetic field^47^. Modern objectives showed up to maximum 6% of measured magnetic field distortion in the image plane of the objective^47^.

### Autofluorescence decay of HeLa cells upon exposure to magnetic field

Cells were seeded in 6-channels Ibidi μ-slides (Ibidi, Germany) and propagated until 80–85% of confluence. After, cell culture medium was replaced with preheated calcium/magnesium free PBS buffer. Cells were washed twice with PBS buffer before the live imaging.

We performed autofluorescence magnetic field measurements in a very similar way to the methodology described in^46^. We focused on HeLa cells utilizing bright-field illumination to avoid photobleaching. Afterwards, cells were imaged under continuous irradiation with 100% power of 488-nm laser embedded in the confocal system and an applied magnetic field varying between either + 10 mT and − 10 mT or + 20 mT and − 20 mT at frequencies of 0.1 Hz or 0.2 Hz. The spinning disk confocal system IXplore SpinSR (Olympus, Japan) is equipped with 100 mW 488 nm laser diode (IX-LAS488-100LSS, OBIS laser, Coherent Corp., US). The laser power was measured with an optical power and energy meter PM100D (Thorlabs Inc., US) using S121C power sensor (Thorlabs Inc., US). The measured power close to focal plane was 58 mW. The irradiated region with 100 × silicone immersion objective is estimated to be ~ 133 µm × 133 µm. Thus we calculated an overall irradiation intensity to be ~ 0.33 kW/cm^2^ on the sample. This intensity is in line with previously reported for spinning disk microscopes^49^. The built-in thermometer inside the Hall probe was additionally used to control the temperature stability or absence of heating effects. Fluorescent images were captured with a camera exposure time of 100 ms.

### High-resolution fluorescent imaging

In order to get high-quality fluorescent images for further autofluorescence decay analysis, the high-resolution spinning disk confocal system IXplore SpinSR (Olympus, Japan) was used. The system utilizes an inverted microscope (IX83; Olympus, Japan) and a spinning disc confocal unit (CSUW1-T2S SD; Yokogawa, Japan). Fluorescence images were obtained through 100 × silicone immersion objective (UPLSAPO100XS NA 1.35 WD 0.2 silicone lens, Olympus, Tokyo, Japan). Autofluorescence was excited by 488 nm laser. Confocal images were acquired at a definition of 2048 × 2048 pixels. A bandpass filter (BA510-550; Olympus, Japan) was used before scientific Complementary Metal Oxide Semiconductor (sCMOS) camera ORCA-Flash4.0 V3 (Hamamatsu, Japan). Images were taken with the acquisition software cellSens (Olympus, Japan).

For cell size and circularity measurements, cells were labelled with CellMask Green (Thermo Fisher Scientific, US) to visualize the plasma membrane. Cell membrane was used as a mask to determine the edges of the cell. Labeled cells were imaged using confocal microscopy. ImageJ software (NIH, US) was used to calculate cell area and circularity.

For 3D reconstruction cells were labeled with CellMask Green (Thermo Fisher Scientific, US), MitoTracker Red CMXRos and hoechst 33342. Labeled cells were imaged by confocal microscopy. An open-source software Icy (https://icy.bioimageanalysis.org)^50^ was used for 3D reconstruction.

To measure our confocal system sensitivity and applicability for autofluorescence measurements, we utilized a laser system described previously^51^. 505 nm laser spot irradiation was performed using taper ~ 15 µm in diameter with irradiation intensity of ~ 0.06 kW/cm^2^. The images were captured with a camera exposure time of 100 ms. ImageJ software (NIH, US) was used to calculate integrated intensity.

### Image processing and data analysis

Fluorescent signal from a single cell (defined by region of interest—ROI) was defined as the sum of pixel intensity for a single image with the subtracted average signal per pixel for a region selected as the background. Such analysis was done using function *intensity profile* in software CellSens (Olympus, Japan). We analyzed autofluorescence decay of 90 to 109 individual cells per condition. We performed three independent experiments on different days. To compare levels of an integrated density, we used ImageJ software (NIH, US).

Curve fitting and residual analysis were done in SigmaPlot 13.0 software (Systat Software Inc., US). Global curve fitting was conducted using a single exponential decay function (*f* = *y0* + a*exp(− b*x)). Normalized residuals were calculated as (obtained value − fitted curve value)/(fitted curve value). Additionally, we calculated MFE, defined as [*I*(*B*_*0*_)* − I*(*0*)]/*I*(*0*), where *I*(*B*_*0*_) and *I*(*0*) are the fluorescence intensities in the presence and absence of the magnetic field respectively^34,37,38^.

### Fluorescent probes

To highlight differences in brightness of synthetic fluorescent probes and endogenous fluorescence, we labelled cells with standard fluorescent dyes. Cells were labeled with CellMask Green (C37608, Thermo Fisher Scientific, US) in order to visualize plasma membrane. Additionally, mitochondria were stained with MitoTracker Green FM (M7514, Thermo Fisher Scientific, US). Stained cells were imaged using the spinning disk confocal microscope IXplore SpinSR (Olympus, Japan). For colocalization analysis, cells were labelled with either LysoTracker Red DND-99 (L7528, Thermo Fisher Scientific, US) or MitoTracker Red CMXRos (M7512, Thermo Fisher Scientific, US) probes and imaged using spinning disk confocal microscopy.

### Fluorescence spectra measurements

The fluorescence spectra of HeLa cells and a solution of Atto488 were measured with a FS5 spectrofluorometer (Edinburgh Instruments Ltd., UK). The excitation was performed with a Xenon-Arc Lamp light with a selected excitation wavelength of 450 nm. The excitation slits and the corresponding light powers were 1 and 5 nm, and 0.06 and 1.2 mW, for the synthetic dyes and for the autofluorescence of the cells, respectively. The fluorescence was acquired from 480 to 800 nm using 1 nm resolution and a dwell time of 0.2 s with 3 times averaging.

### Statistical analysis

The sample size determination was assessed utilizing a statistical method described in^52^, taking into assumption 95% confidence level and 0.9 statistical power. The statistical significance of differences between the groups was determined using ANOVA with subsequent application of Dunnett’s test. All statistical analyses were performed using MaxStat Pro 3.6. Differences were considered statistically significant at (*) *P* < 0.05.

For a quantitative image assessment, we used the published guidance for quantitative confocal microscopy^53,54^. Images from three independent experiments were subjected to quantitative analysis. In each experiment, at least 90 cells from each sample were subjected to quantitative analysis.

## Results

### Size and shape of HeLa

Studies of the effects of magnetic fields on living cells represent an interesting and very challenging task. There have been many attempts to directly measure MFEs and link them to RPM. Current literature enumerates many studies from various cell-free enzyme systems. However, not all MFEs were shown to be reproduced independently^11,25,26,28,29,35,55^. Therefore, we wanted to contribute to the existing knowledge in this field by analyzing currently published observation of MFE on autofluorescence of HeLa cells^46^. Part of current study was deposited on the preprint server^56^. The authors of the original study have replied with another preprint^57^ bearing additional information and a critical assessment of our re-evaluation study. We found these comments very useful and interesting, adding important points which we would like to further unfold.

**Figure 1:**
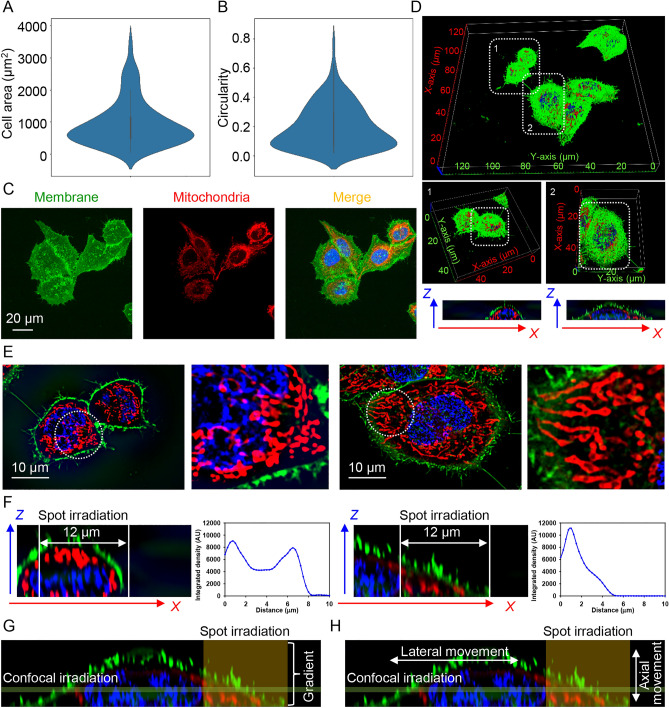
Size and geometry of HeLa cells. HeLa cells were labelled with CellMask Green (Thermo Fisher Scientific, US) in order to visualize plasma membrane. Mitochondria were labelled with MitoTracker Red CMXRos (Thermo Fisher Scientific, US). Hoechst 33342 was used as a counterstain for nucleus (blue). Labeled cells were imaged by confocal microscopy. ImageJ software (NIH) was used to calculate cell area (**A**) and circularity (**B**). N = 147 individual cells were assessed. Violin plots were created using open-source software (http://www.bioinformatics.com.cn/login_en/). (**C**) Example of imaged HeLa cells labelled with CellMask Green, MitoTracker Red CMXRos and hoechst 33342 (blue). Labeled cells were imaged by confocal microscopy. (**D**) HeLa cells were labeled as in (**A**), imaged by confocal microscopy to acquire 3D optical sectioning with z-interval of 250 nm. Optical sections were deconvolved using CellSens software (Olympus, Japan). 3D reconstruction was done using open-source software Icy (https://icy.bioimageanalysis.org). *Z–X* projections of selected cells were done using ImageJ software (NIH, US). (**E**) Confocal plane images of selected cells from (**D**). Dotted circle represents hypothetical laser spot illumination of ~ 12 µm in diameter. (**F**) *Z–X* projection of selected cells from (**D**). *Z–X* projection illustration of hypothetical laser spot illumination of ~ 12 µm in diameter. Graphs represent of *Z*-axis profile measurement of MitoTracker Red CMXRos intensity. Graphs were created with SigmaPlot 13.0. (**G**) Schematic representation of differences between confocal and spot irradiation of cells. (**H**) Schematic representation of lateral and axial movements of organelles within a cell.

The authors of the original study^46^ and the preprint^57^ insist that it is crucial to have spot irradiation of individual HeLa cells. They selected a spot with a diameter of 12 μm^57^. This selection is based on the average size of HeLa cells creating a spot that irradiates single-cell subcellular region without affecting the nucleus and neighboring cells. The average HeLa cell area as measured by confocal microscopy represents 943 µm^2^ average with broad distribution (Fig. 1A). This area can be roughly recalculated to 17.3 µm, with the average size of HeLa cells, ranging from 15 to 33 µm in accordance with cell area measurements (Fig. 1A). These findings are in line with previous size measurements of HeLa cells, e.g. average HeLa cell size measured to be 17.1 µm^58^. The area measurement data clearly indicates that size distribution of individual HeLa cells is heterogenous (Fig. 1A). The cell size range is clearly bigger than the selected spot irradiation diameter in the preprint^57^. Further, we performed analysis of cell circularity. It is evident that the majority of HeLa cell population bears low circularity lower 0.2 (Fig. 1B). This clearly shows that individual HeLa cells are not circular but of various shapes. In fact, this was confirmed by direct HeLa cell shape observation (Fig. 1C).

Measurements of cell size and cell shape analysis imply that the degree of individual cell irradiation will ultimately vary dramatically between even neighboring cells. In addition cells are 3D objects that possess different sizes in 3D. One can clearly see differences in individual cell sizes of neighboring cells reconstructed in 3D (Fig. 1D). We zoomed in on two neighboring cells from the same field of observation (Fig. 1E). One can see how different spot irradiation would be related to cell size (Fig. 1E). In one case spot irradiation takes the majority of the cell, in the other—only fraction of the selected cell (Fig. 1E). These differences become even more evident in 3D with *Z–X* projection (Fig. 1F). Spot irradiation of a small cell will inevitably result in nucleus irradiation (Fig. 1F). On the contrary if a bigger cell is exposed to spot irradiation, then the nucleus may well be avoided (Fig. 1F). Cell size heterogeneity is connected with heterogeneity of organelles distribution within the cell. Thus, one can see that for example mitochondria (according to study^46^ major source of FAD molecules responsible for MEFs) possess different volume distribution in two neighboring cells (Fig. 1F). Further, we would like to schematically illustrate that spot irradiation of a cell will result in non-even light distribution within the entire cell when confocal irradiation leads to more even photoexcitation (Fig. 1G). It is evident from Movie S1 in the original study^46^ illumination leads to way broader photoexcitation then the spot itself. The authors correctly state in the preprint^57^ that upon confocal illumination we may lose autofluorescent entities due to axial movement (Fig. 1H). However, spot irradiation that does not cover the whole cell suffers from a similar problem. Under such irradiation conditions there is a high probability of losing the signal due to lateral movement of autofluorescent entities (Fig. 1H). This movement is directly visible in Movie S1 from the original study^46^.

Our analysis shows that both spot and confocal irradiation bears pros and cons in illuminating cells and therefore are interchangeable. Importantly, cell size and shape show dramatic heterogeneity within HeLa cell population. Additionally, organelle volume distribution (in particular mitochondria) is also heterogeneous. All these facts together strongly indicate that for getting reliable autofluorescent measurements, the appropriate sample size and statistical power must be applied to avoid false positive results.

Further, in the preprint^57^ authors indicate that they prepared cells for autofluorescence measurements in a particular way. They propagated cells, detached, seeded to appropriate microscopy compatible slide/chamber, gave rest, and before measuring the autofluorescence washed cells with PBS replaced medium and imaged in PBS to avoid background^57^. We followed the same procedure. Importantly, the authors in the preprint indicate that cells were left in the chamber for 42 to 48 h to recover from stress^57^. The doubling time of HeLa cells was measured as 33–35 h^59^. This indicates that after 42 to 48 h the HeLa cell population seeded on slide/chamber will bear cells at different cycle phases. Importantly, evidence suggests that flavins’ (in particular FAD) intracellular concentration dramatically fluctuates depending on the cellular metabolic status and cell cycle phase^60–62^. These facts strongly support our above given conclusion that appropriate sample size and statistical power must be applied to single cell autofluorescent measurements.

### The irradiation conditions

Justification and explanation of the irradiation conditions are presented in previous studies^46,57^. Specific irradiation conditions generate the fluorescence signal from cells, and drive the magnetically sensitive photocycle^57^. However, no specificity and reasoning for crucial parameters of photoexcitation are discussed, e.g. light wavelength, power, irradiance, pulse duration^57^.

We performed systematic analysis of the irradiation conditions utilized in the original study^46^, preprint^57^ and other studies that detected magnetic field effects and/or radical pair formation of flavin adenine dinucleotide cell-free solutions (Table 1). From this table it is clear that the irradiation conditions vary dramatically. This systematic analysis reveals that the wavelength of the light used for photoexcitation varies from 355 to 488 nm (Table 1). The pulse length for pulsed photoexcitation ranges from 2–3 ns to 1 s (Table 1). This is an enormous range (about 9 orders of magnitude). Continuous-wave excitation was used as well (Table 1). The frequency employed also varies from 0.14 Hz to 10 kHz (Table 1). Additionally, there appears to be no consistency in light power used for photoexcitation (Table 1). With powers used ranging from 1 mW to 1000 W (Table 1). This huge difference in the photoexcitation conditions suggests that there are probably no specific photoexcitation conditions required to observe MFE in FAD cell-free solutions. Absorbance range of the endogenous fluorophores varied from 330 to 490 nm (Table S1). Specifically, FAD and FMN excitation peak position range was from 380 to 490 nm (Table S1). Thus, considering the enormous range in photoexcitation parameters in the studies where MFEs of flavin adenine dinucleotide cell-free solutions were observed (Table 1), we can reasonably conclude that the irradiation conditions are not strictly predisposed. The irradiation wavelength should fall in the range of FAD excitation peak position, the irradiance may be of approximately same value as in the study^46^. The pulse length, power or frequency do not represent important parameters because they vary orders of magnitude in different studies (Table 1). Moreover, it has been shown that photoexcitation with 488 nm laser result in RPs formation in FAD solutions^63^. Further, it is indicated that irradiation should be suitable enough to generate the autofluorescence from cells, and support the magnetically sensitive photocycle, i.e. formation of RPs^57^. Therefore, we concluded that using 488 nm laser excitation in spinning disk confocal system is a valid system to study MEFs in HeLa cells. Such conditions provide enough power to induce autofluorescence and support RPs formation of FAD molecules^63^.

**Table 1:** Summary of the irradiation conditions from literature that shows magnetic field effects and/or radical pair formation of flavin adenine dinucleotide cell-free solutions.

Light irradiation conditions						References
Wavelength (nm)	Irradiance (kW cm^−2^)	Pulse length	Frequency	Irradiation source (producer)	Light power	
450	1.0	5 ms	100 Hz	CW diode laser (PLT5 450B, OSRAM)	1.0 mW	^57^
450	0.5	Continuous	100 Hz	CW diode laser (PLT5 450B, OSRAM)	1.0 mW	^46^
449	N/I	700 ns	10 kHz	Pulsed laser (CUBE laser, Coherent)	~ 2.0 mW	^34^
473	N/I	Continuous	N/A	CW laser (N/I)	N/I	^37^
355	N/I	2–3 ns	5 Hz	Pulsed Nd:YAG Laser (Spectra Physics GCR-3)	15 mJ per pulse	^37^
405	N/I	Continuous	N/A	CW diode laser (Power Technology)	350 mW	^38^
355	N/I	2–3 ns	N/I	Pulsed Nd:YAG Laser (Spectra Physics GCR-3)	15 mJ per pulse	^102^
N/I	N/I	1 s	2 Hz	Mercury lamp (SP lOOOWQ, Philips)	1000 W	^103^
405	N/I	Continuous	N/A	CW diode laser (Power Technology)	350 mW	^104^
488	N/I	0.4 s	0.14 Hz	Pulsed argon ion laser (Spectra Physics 171)	10 W	^63^
355	N/I	2–3 ns	N/I	Pulsed Nd:YAG Laser (Spectra Physics GCR-3)	N/I	^105^

*N/A* not applicable, *N/I* not indicated, *CW* continuous-wave.

### Autofluorescence of HeLa cells and quantitative fluorescence microscopy

Concept of MFE at cellular level is based on the hypothesis that an external magnetic field affects the photochemistry of flavins^46^. Specifically, it can influence the spin-correlated radical pairs formed via intersystem crossing in the excited state of a flavin molecule. This would change the rate of flavin deexcitation and the concentration of flavin molecules in the ground state. As a result, a change in the cellular endogenous fluorescent signal should be observed under continuous photoexcitation^46^. In other words, the autofluorescence photobleaching should be altered by external magnetic field. Furthermore, it was proposed that flavins are the major source of cellular autofluorescence in the range 480–650 nm when excited with blue laser (450 nm)^46^.

**Figure 2:**
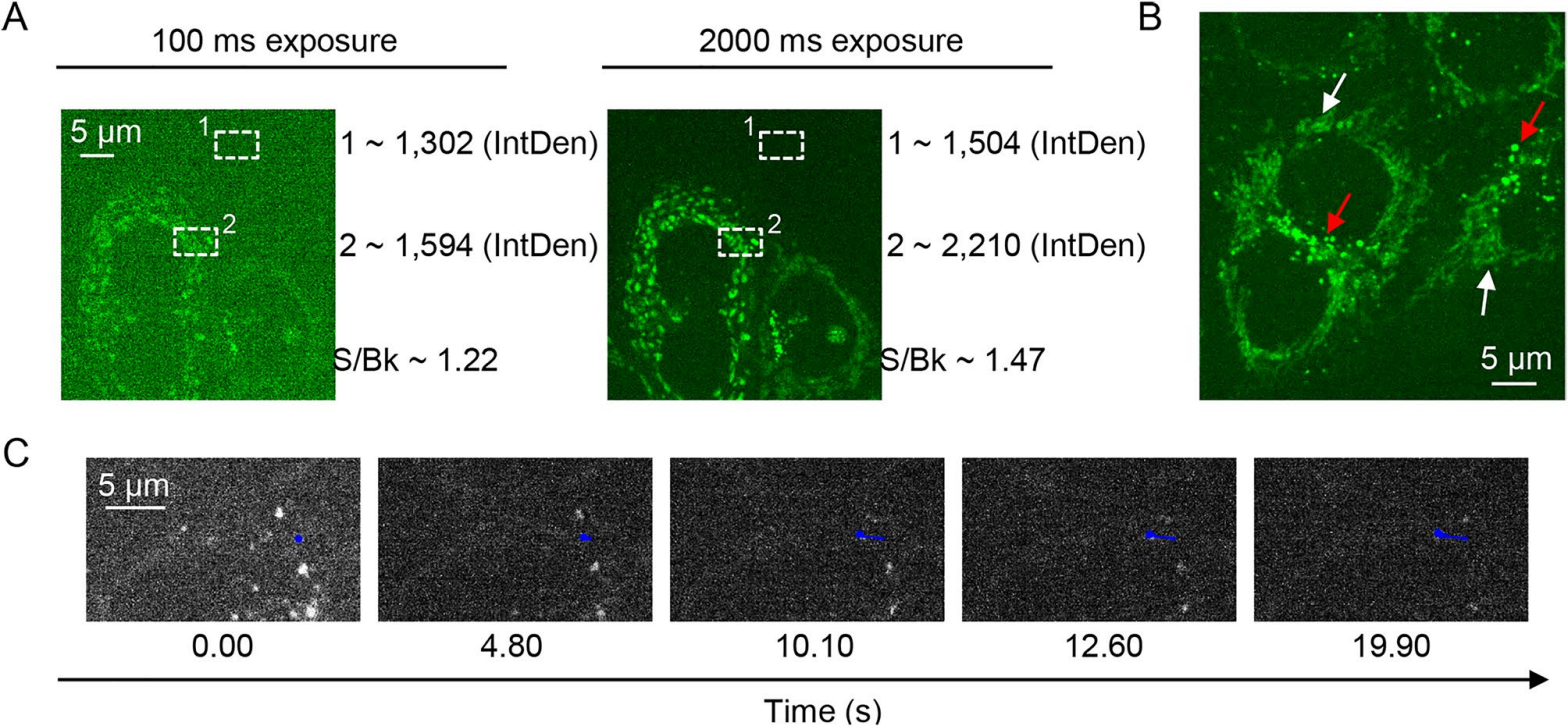
Autofluorescence of HeLa cells. (**A**) Autofluorescence of HeLa cells at 100 ms and 2000 ms exposure time. Integrated density (*IntDen*) was measured for the background (1) and cell region (2) using ImageJ software (NIH, US). Signal-to-background ratio (*S/Bk*) was calculated for image selections. (**B**) Enlarged picture represents tubular (white arrows) and round (red arrows) intracellular structures. 2000 ms exposure time. (**C**) Vesicle tracking within the time range, 0–20 s. Blue color represents vesicle movement through the time-lapse image (Movie S3).

Healthy undamaged cells possess relatively low level of autofluorescence in comparison to modern synthetic exogenous fluorophores^64–66^, which are nowadays greatly optimized to offer higher quantum yield and brightness (Table S2). In fact, HeLa cells imaged in phosphate-buffered saline (PBS) showed relatively dim autofluorescence (Fig. 2A and Fig. S3). We utilized the similar conditions for excitation (“blue” 488 nm laser) and emission detection (BA510-550 nm “green” filter and sCMOS camera ORCA-Flash4.0 V3) as in the study^46^. In the previous section we explained in great detail why such incremental differences in photoexcitation conditions will not impact on RP formation and autofluorescence measurements. We as well provided our rationale why spot irradiation proposed in the original study^46^ is not a crucial parameter for the observation of MFEs in cells. Thus, we irradiated all cells captured in the field of view to increase the sampling size. Actually, the electromagnets used in the original study and in ours are relatively bulky, which precludes focusing of the magnetic field on a single cell. Since all cells on the microscopy slide are subjected to the same magnetic field, we illuminate all of them in order to get more samples for a better statistical assessment of the results. It was noted that cells, generally, showed a magnetic response with a magnitude of 1 to 2.5%^46^. These are very small changes, that we think require large sampling for reliable statistical assessment.

Guidance for quantitative fluorescence microscopy state that for a change of even as high as 25% between two conditions, sampling of ~ 100 cells for each condition is required to measure the change with statistical confidence^53^. When analyzing the data for autofluorescence decay, we performed a background correction. It is absolutely necessary and essential for accuracy and precision in quantitative fluorescence microscopy measurements, especially when quantifying weak signals^53,67^.

Further analysis of HeLa cell autofluorescence revealed that one can get better imaging using a longer (2 s) exposure time (Fig. 2A and Fig. S3). This is typical in the detection of dim fluorescent signals^53,67^. Interestingly, there are various endogenous fluorophores that contribute to “green” (500–550 nm) autofluorescence of cells^64,66^, for details see Supporting Information, Table S1. Not only flavins are responsible for such autofluorescence, but also lipofuscin and free fatty acids (Table S1). Figure 2B and Fig. S4 show that “green” autofluorescence originates from at least two distinct subcellular structures, i.e., tubular, and vesicular. In fact, the same (tubular and vesicular) structures are clearly visible in Movie S1 from the original study^46^. The main subcellular structures contributing to “green” autofluorescence are the mitochondria and lysosomes^68,69^. Autofluorescence from mitochondria was proposed to come from flavins (for example, FAD), whereas lysosomal autofluorescence is presumably due to lipofuscin accumulation^68,69^. Taking together those studies and our results (Fig. 2B), we can say that it is not a single subcellular structure contributing to the autofluorescence. Taking into account the observed autofluorescence of HeLa cells is weak (Fig. 2A) is important for quantitative analysis of the images, due to Poisson noise persistence in fluorescence microscopy digital images^67^. For example, bright synthetic exogenous fluorophores provide better suitable images (Fig. S5) for quantitative analysis in contrast to autofluorescence (Fig. 2A).

From the captured autofluorescence photobleaching movie (Movie S1) it is clear that the autofluorescent structures are moving. To present moving structures more clearly, we zoomed in several cells (Movie S2). Additionally, we performed particle tracking analysis of selected vesicles (Fig. 2C and Movie S3) and found them moving approx. 2.5 μm in 20 s (Fig. 2C and Movie S3). We think that weak fluorescence signal in combination with fast-moving structures may add disturbance in further quantitative image analysis^53,67^. Moreover, different cells display different levels of autofluorescence intensity (Fig. S6). As a result, autofluorescence decay upon photobleaching in different cells largely varies (Fig. S7 and Movies S4 and S5). Further, some cells contain both highly movable and non-movable fluorescent entities (Fig. S7 and Movie S6).

**Figure 3:**
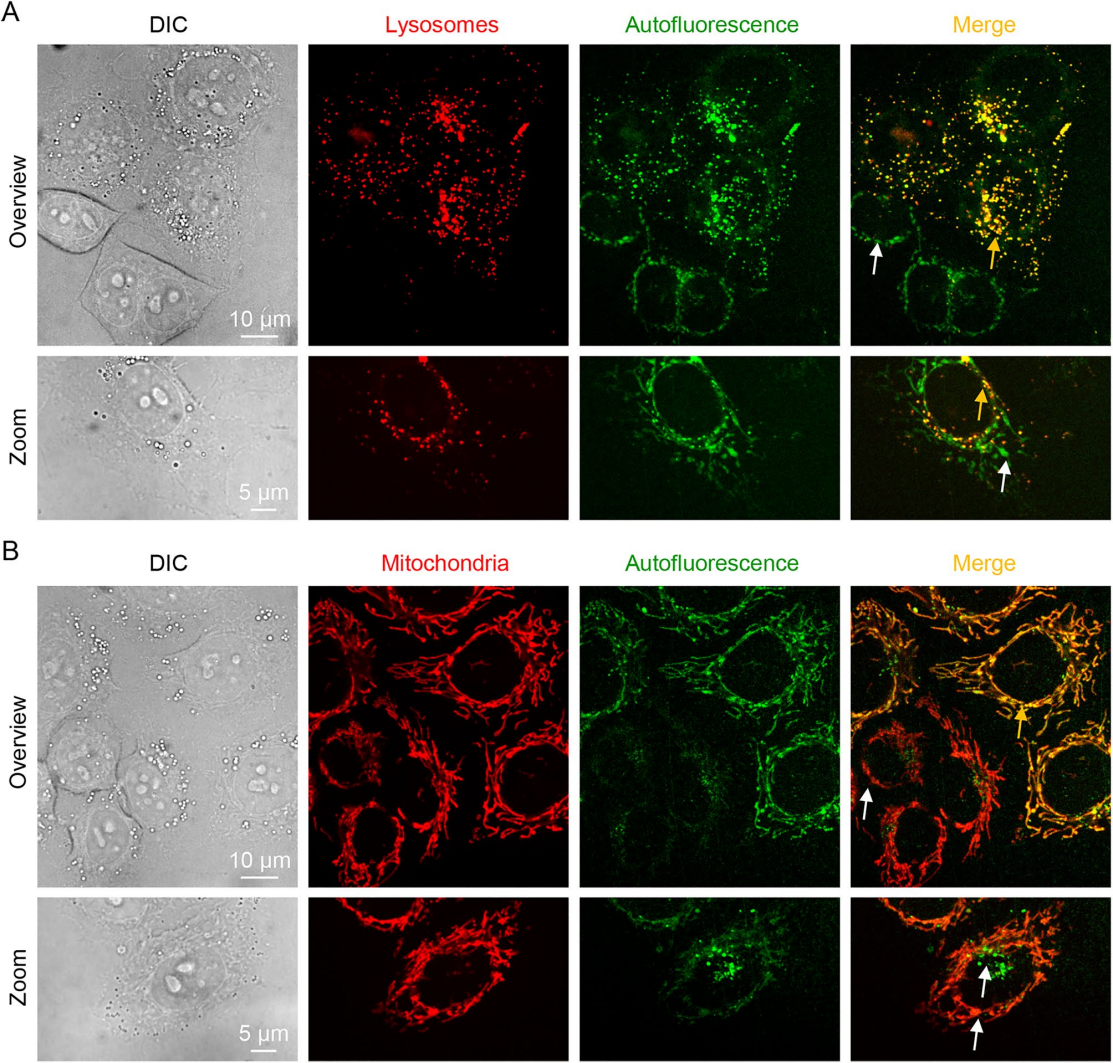
Colocalization of autofluorescence signal in HeLa cells. (**A**) Cells were labeled with fluorescent probe LysoTracker. Labeled cells were imaged by confocal microscopy for LysoTracke (100 ms exposure time) and autofluorescence signal (2000 ms exposure time). The digital images were processed using ImageJ software (NIH). Yellow arrows show colocalization of autofluorescent signal with lysosomal probe. White arrows indicate no colocalization of autofluorescent signal with lysosomal probe. (**B**) Cells were labeled with fluorescent probe MitoTracker. Labeled cells were imaged by confocal microscopy MitoTracker (100 ms exposure time) and autofluorescence signal (2000 ms exposure time). The digital images were processed using ImageJ software (NIH). Yellow arrows show colocalization of autofluorescent signal with mitochondrial probe. White arrows indicate no colocalization of autofluorescent signal with mitochondrial probe.

To validate further different origins of autofluorescent signal in HeLa cells, we performed colocalization analysis. We labeled cells with LysoTracker and MitoTracker probes and imaged labeled cells using spinning disk confocal microscopy (Fig. 3). As we already mentioned, this imaging revealed that autofluorescence is heterogeneous and highlights two distinct subcellular structures, i.e., tubular, and vesicular (Fig. 3). Colocalization analysis using LysoTracker probe reveals that not all autofluorescent structures colocalize with lysosomes (Fig. 3A). Similar pattern we found with MitoTracker probe—not all autofluorescent structures colocalize with mitochondria within a single cell (Fig. 3B). These data indicate that autofluorescent signals within a single cell originate from at least two distinct sources e.g., lysosomes and mitochondria. It has been shown that lysosomal autofluorescence is predominantly formed by lipofuscin accumulation^68,69^.

Of note, it is still not precisely verified what endogenous fluorophores contribute to cellular autofluorescence^70^. However, main contributors to autofluorescence of 500–600 nm range were identified as NAD(P)H, flavins, ceroid/lipofuscin and bilirubin^70^. Importantly, lipofuscin bears similar brightness as FAD (Table S2). Therefore, we think that it is not justifiable to neglect lipofuscin as a source of cellular autofluorescence^46^. Summarizing, one can conclude that the total cell autofluorescence has mixed origin and is formed not only by flavins. Seemingly, in the light of all above mentioned, measurements of “green” autofluorescence decay are highly variable. In addition, distinct chemical entities (presumably flavins and lipofuscin) contribute to cell autofluorescence.

### MFE measurements in HeLa cells

We intended to conceptually verify the observations that the change of average fluorescence intensity corresponds to the frequency of the externally applied modulated magnetic field^46^. Thus, we selected amplitudes (± 20 and ± 10 mT) and frequencies (0.1 and 0.2 Hz) of magnetic field. Colocalization analysis confirms that autofluorescent signals within single cell originate from multiple organelles (Fig. 3). Additionally, we illustrated that levels of autofluorescence intensity vary between different cells (Fig. S6). Autofluorescent structures can move at relatively high speed (Fig. 2C and Movie S3). All these factors may contribute to the large variability in the observed autofluorescence decay upon photobleaching (Fig. S7 and Movies S4 and S5), and this observed variability will spill over into the quantitative analysis of images.

It is critical to estimate the minimum number of replicates required for multilevel regression to avoid bias. Importantly, only large effects can be detected using small sample size^71^. A small sample size can lead to overestimates of effect and importantly low reproducibility of results^72^. We think that the observed magnetic response of cells in the order of 1 to 2.5% is a relatively small effect^46^. For such a small effect, we believe, it is important to have at least 100 replicates in order to obtain sufficient statistical power, a prerequisite required for obtaining significance^73^. Therefore, in our study we analyzed autofluorescence decay of 90–109 individual cells per condition (Figs. S8 and S9). However, only performing replicates is not enough; repeat experiments should be conducted on different days^74^. Therefore, we performed three independent experiments on different days (Figs. S8 and S9). The autofluorescence measurement were done using CellSens software (Olympus, Japan). As can be seen the autofluorescence decay (Fig. S8) is very variable within a cell population, even in controls (without magnetic field). Some cells, even without applied field, show fluctuating “spikes” of autofluorescence during photobleaching course (Figs. S8 and S9). In addition, a closer investigation at individual cell autofluorescence changes (Figs. S8 and S9) indicate that there is no pattern changed upon application of magnetic field of different amplitude and frequency. Of note, non-oscillating SMF exposure results in similar “spikes” of autofluorescence as oscillating field (Figs. S8 and S9). The presence of “spikes” indicates that there is a great variability in autofluorescence signal in population of HeLa cells that comes from variable sources of autofluorescence, low autofluorescence signal intensity, rapid movement of autofluorescent entities. Importantly not all cells possess “spikes” (Figs. S8 and S9), they occur randomly in all groups, e.g. control, SMF, oscillating magnetic field (Figs. S8 and S9).

**Figure 4:**
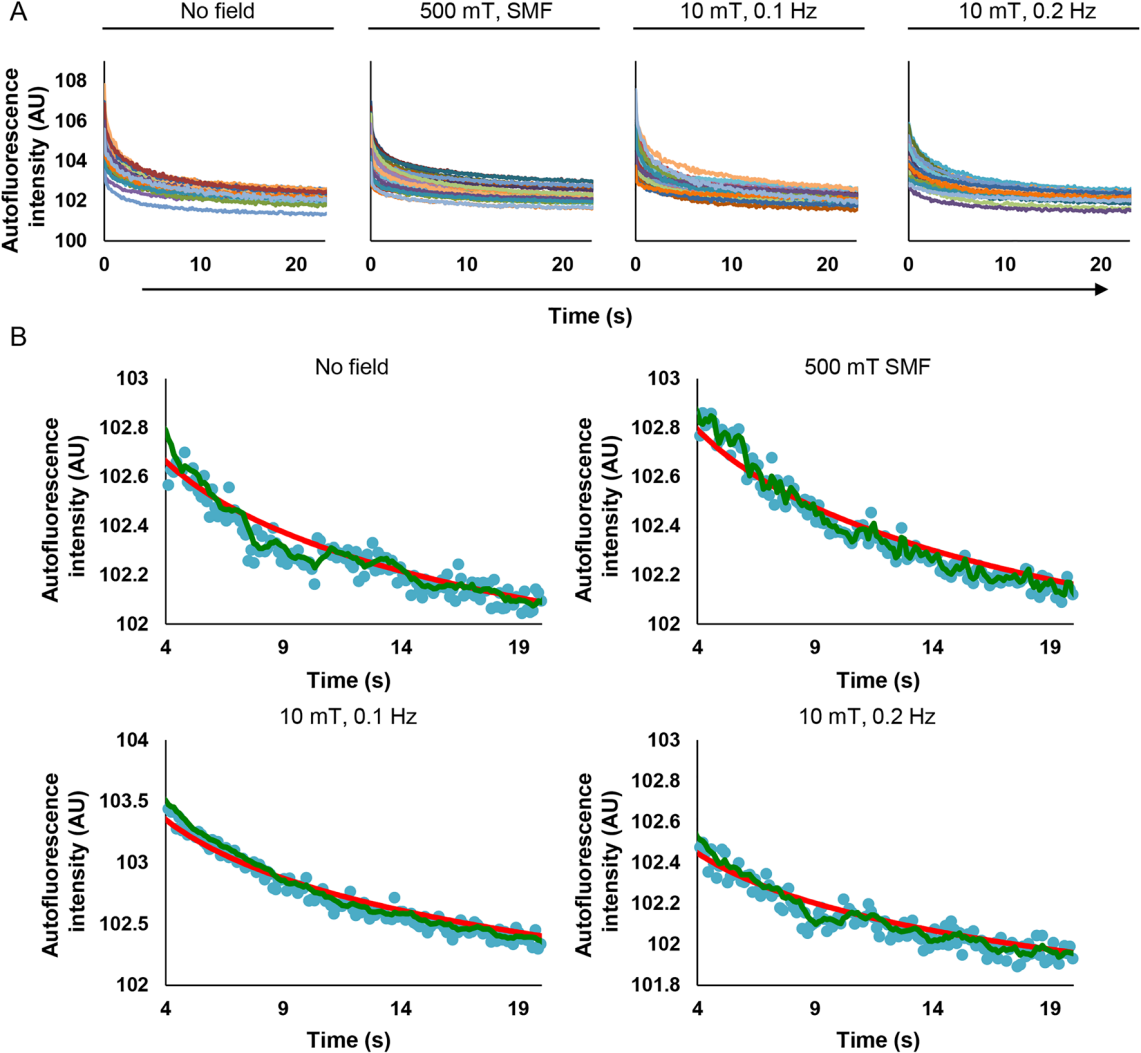
Autofluorescence decay of single HeLa cells upon a magnetic field exposure. (**A**) Autofluorescence intensity within time-frame of 20 s of 25 cells for control (without magnetic field exposure), bulk magnet (500 mT) exposure and a modulated magnetic field 10 mT (frequencies 0.1 Hz and 0.2 Hz). Autofluorescence is presented as mean gray value as assessed by ImageJ software (NIH). (**B**) Examples of single cell autofluorescence decay assessed as in (**A**). Fitting of autofluorescent decay was done by using either exponential trendline (red line) or moving average trendline (green line). All graphs were created with Excel 365.

In order to analyze changes in autofluorescence in more details, we selected 25 random cells from each treatment condition (Fig. 4A). We added one more control, that is a static magnetic field (SMF), generated by a bulk magnet. SMF was applied perpendicular to the sample cover glass. Assuming the cell autofluorescence being magnetic field dependent, we tentatively hypothesized that a static magnetic field of strength of an order of magnitude higher than oscillating one would impact on end stage of biochemical reactions. Thus, in turn such effect will spill over into a change in the autofluorescence decay. Moreover, SMFs of 0.2–1 T were used in many studies revealing magnetic field effects on chemical reactions in a test tube in vitro and showed marked influence on rate of various chemical reactions, for review see^3,75^ and references therein. Further, we intended to have a non-oscillating magnetic field control to correlate a potential impact of oscillating magnetic field with oscillations in autofluorescence signal. Therefore, we added SMF exposure as an additional control treatment to validate whether autofluorescence of HeLa cells is sensitive to a magnetic field. Importantly, we performed averaging of the autofluorescence on a single cell level utilizing mean gray values as the autofluorescence measured by ImageJ software. We based our calculations on the availability of information presented in^46^. The autofluorescence decay curves of 25 randomly selected cells showed considerable variability between single cells (Fig. 4A). Under the experimental conditions used in this study, we were unable to detect noticeable fluorescence changes corresponding to the frequency of the applied modulated magnetic field (Fig. 4A). Further, we fitted experimental data to an exponential function (red line) for the period between 4 and 21 s (Fig. 4B). The experimental data points (blue points) fluctuated on random over fitted curve including control cell where no magnetic field was applied (Fig. 4B). These altogether indicate that under the experimental conditions used in this study, it was unlikely to detect fluorescence changes corresponding to the frequency of the applied modulated magnetic field (Fig. 4B). Additionally, we also performed moving average fitting (green line) of the experimental data (Fig. 4B). Once again, we found no dependency of fluorescence changes corresponding to the frequency of the applied modulated magnetic field (Fig. 4B).

**Figure 5:**
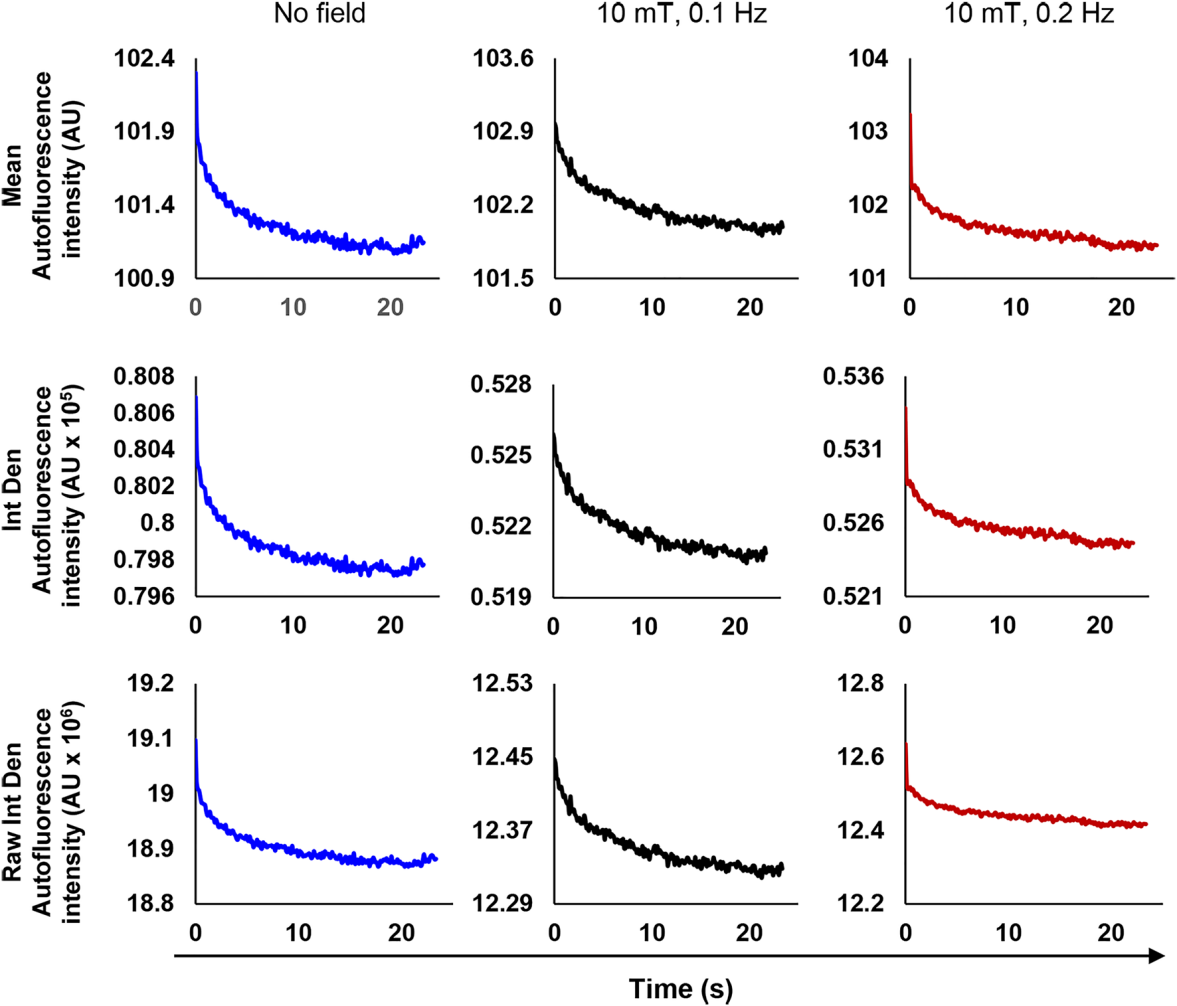
Single cell averaged autofluorescence decay of cells presented as *mean gray value* (Mean), *integrated density* (Int Den), and *raw integrated density* (Raw Int Den). Autofluorescence decay of cells as assessed by ImageJ software (NIH). All graphs were created with Excel 365.

It is worth noting here that default measurement settings of ImageJ software derive average value of the image parameters corresponding to the fluorescence signal over selected ROI (page 136^76^). Parameters that are used for quantitative analysis of the fluorescence signal are *mean gray value* (basically averaging of fluorescence over selected ROI), *integrated density* (the product of *area* and *mean gray value*), and *raw integrated density* (sum of pixel values)^76^. Indeed, one can clearly see that all three parameters are equivalent showing the same shape of autofluorescence decay (Fig. 5, Figs. S10, S11 and S12). The difference is in absolute values; however, the range of fluorescence decay stays the same for individual cells (Fig. 5, Figs. S10, S11 and S12). Based on the availability of information presented in^46,57^, we tentatively hypothesized that standard averaging of the signal using either *mean gray value* or *integrated density* was done. Importantly, quantification of autofluorescence decay using averaging of the signal from single cell showed that fluorescence change does not correspond to the frequency of the applied modulated magnetic field sweep (Fig. 5, Figs. S10, S11 and S12). In fact, quantification of autofluorescence decay utilizing CellSens software as well does not confirm effect of the magnetic field on the decay trend (Figs. S8 and S9). It is worth noting here, that variability of autofluorescence between different individual cells is huge (Fig. 4, Figs. S8 and S9). Therefore, it is important to perform sufficient number of biological replicates to make correct inference about the mean and variance of a biological population^77^. Doing only technical replication (the same cell serves as a control and treatment) is an approach that makes obtained results exposed to error and unreliable^77^.

### The sensitivity of the optical system

**Figure 6:**
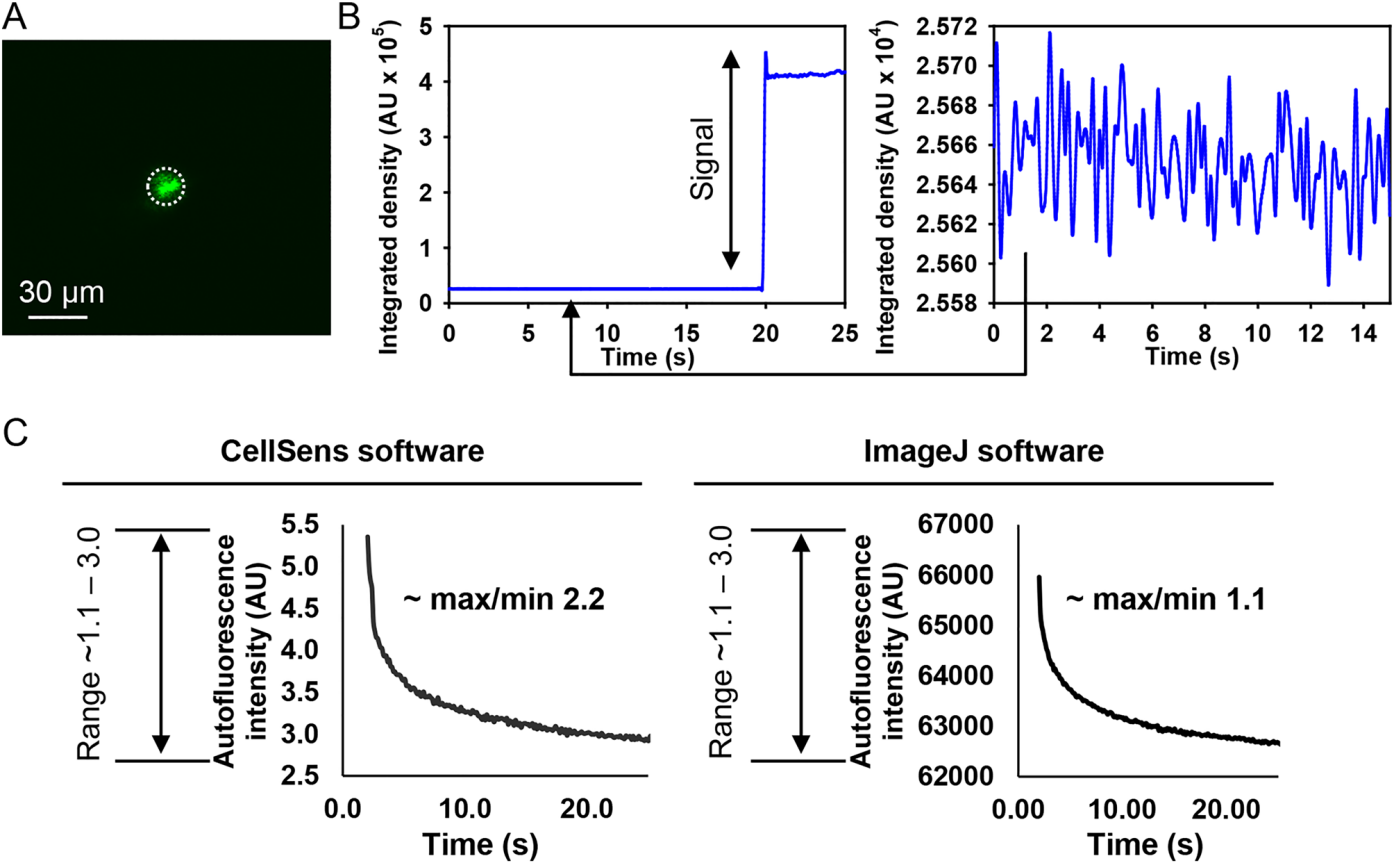
Signal-to noise analysis of the imaging system. (**A**) 505 nm laser spot irradiation was performed using taper ~ 15 µm in diameter. Dotted circle highlights the region of signal integration where intensity within time-frame of 25 s was assessed by ImageJ software (NIH). (**B**) The integrated density changes during the measurement as assessed by ImageJ software (NIH). The zoom-in data shows camera noise. (**C**) Representation of ranges of autofluorescence decay of cells as assessed by ImageJ software (NIH) and CellSens software (Olympus, Japan). Graphs were created with SigmaPlot 13.0.

One may argue that our autofluorescence system is not that sensitive enough to detect small changes in autofluorescence decay^46,57^. We used a 488 nm laser diode (IX-LAS488-100LSS, OBIS laser, Coherent Corp., US) for photoexcitation that wavelength enables RP formation in FAD molecules^63^. A bandpass filter (BA510-550; Olympus, Japan) was used to detect autofluorescence. The crucial part of fluorescence detection in cells is a detector^53,54^, sensitivity of which determines sensitivity of measurements. We used exactly the same detector as in the original study^46^, namely scientific Complementary Metal Oxide Semiconductor (sCMOS) camera ORCA-Flash4.0 V3 (Hamamatsu, Japan). Importantly, we measured, as the authors did in their preprint^57^, the signal-to-noise ratio and noise of our detector (Fig. 6A,B). Of note, the signal-to-noise ratio and camera noise (Fig. 6A,B) were nearly identical to those observed by the authors in the preprint^57^.

Importantly, the authors in the preprint^57^ state that signal-to-noise ratio of their fluorescence signal is high (> 4000 at time of weakest signal). This statement is somewhat perplexing, as software analyzing fluorescent images do not calculate precise autofluorescent signals; rather it calculates either the integrated or raw greyscale intensity of the image, depending on software settings. These intensities are expressed in arbitrary units. Therefore, it is crucial to see the range of changes and not absolute values. For example we processed autofluorescence decay of two different cells using different softwares (Fig. 6C). One can see that the absolute numbers differ, but the range stays the same (Fig. 6C). The range of maximum-to-minimum ratio of autofluorescence stays in the frame of 1.1–3.0 (Fig. 6C). The same as we observed in individual single cells autofluorescence decays (Figs. S8 and S9). This same range was observed by authors in the original study^46^ and in the preprint^57^.

It is important to note that the noise of the image is determined by the variation in output when given a constant input signal^78^. The noise will be at first instance predisposed by the quality of the detector. The background on a resultant image is characterized by the offset in signal intensity equally redistributed over the whole image^78^. A simple example of the background source is stray light (e.g. from illumination source itself) that reaches the detector^78^. Therefore, for low-fluorescent intensity imaging it is crucial to select the appropriate detector^53,54^. It is also extremely important to analyze the range and not absolute numbers of integrated intensity on the resultant image. That is why bright synthetic exogenous fluorophores (Fig. S5) provide reliable images with high signal-to-background ratio for quantitative assessment in contrast to autofluorescence images (Fig. 2A). These facts together, in our opinion, clearly show that a weak fluorescent signal coming from cells and not imaging or detection system is a source of great variability in the data of autofluorescent decay.

### Importance of statistical power and sample sizes in the assessment of cell autofluorescence

**Figure 7:**
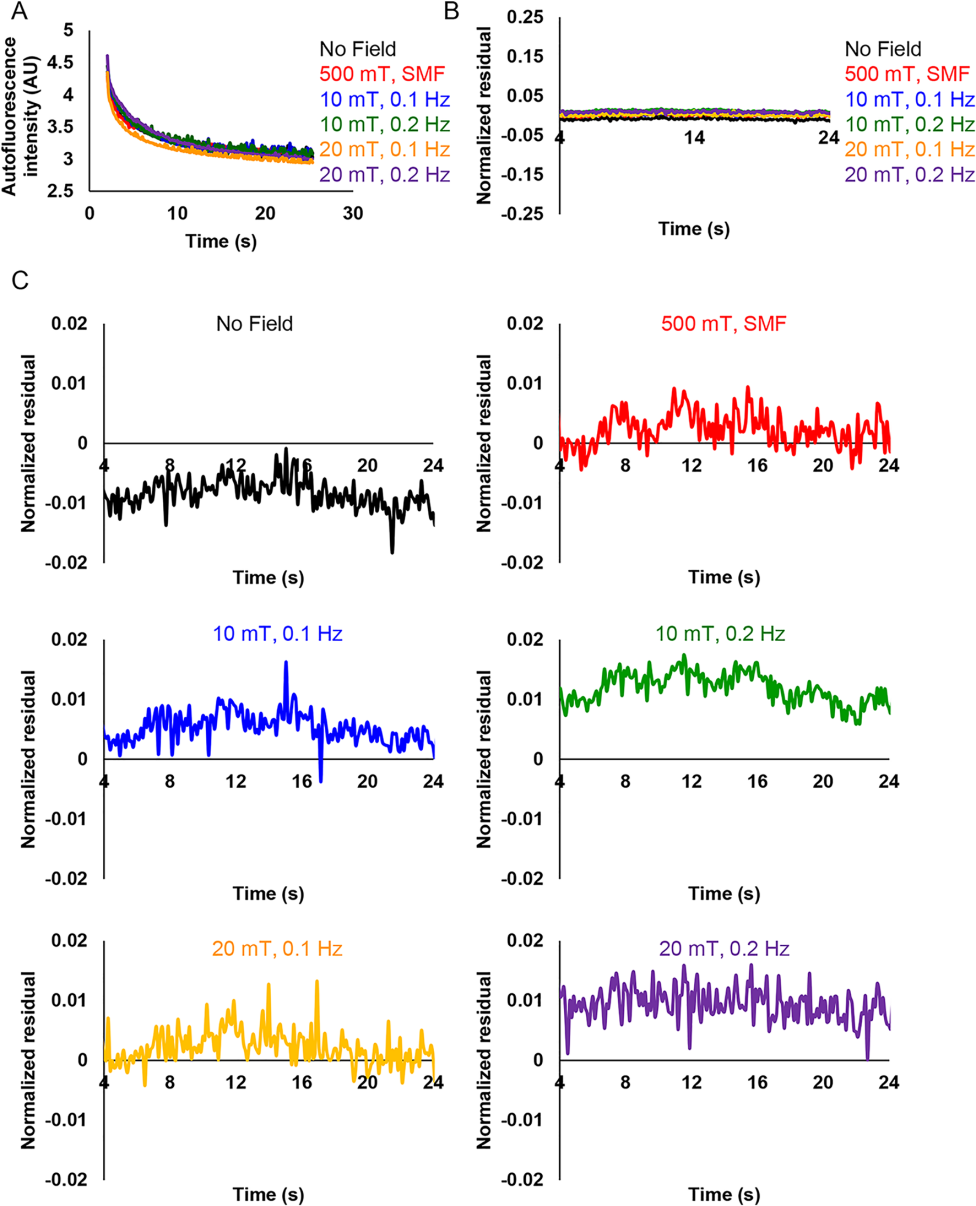
Averaged autofluorescence intensity change of HeLa cells. (**A**) Average autofluorescence decay upon different magnetic field exposure. Cells were irradiated by 10 mT and 20 mT modulated magnetic field (frequencies 0.1 Hz and 0.2 Hz). 500 mT static magnetic field (SMF) was generated by bulk NdFeB magnet. N = 90–107. (**B**) Normalized residuals calculated from the average autofluorescence intensity divided by the obtained intensity from the fitted curve values within 20 s. N = 90–107. (**C**) Normalized residuals from (**B**) presented as separated graphs with zoomed-in Y-axis. All graphs were created with Excel 365.

To illustrate the importance of statistical power and sample sizes in mitigating false positive results, we performed analysis of autofluorescent decay curves from 90 to 105 individual cells. Consistent with the above discussed results, averaging autofluorescent decay curves from 90 to 105 individual cells (Fig. 7A and Fig. S13) did not provide any pattern in the autofluorescence decay. We did not observe a clear fluorescence change corresponding to the frequency of the applied magnetic field (Fig. 7A and Fig. S13). Therefore, we conclude that we observed no effects in the decay of autofluorescence under any of the conditions used (Fig. 7A and Fig. S13). Plotted on the same graph averaged autofluorescence intensity curves look very similar (Fig. 7A).

It is worth noting here that averaged autofluorescence decay curves can be nicely fitted using either single exponential or double exponential 5 parameters decay functions (Fig. S14). Double exponential decay 4 parameters function showed a bad approximation of the data, reflected by low values of R-square and Adjusted R-square, as well as high error value (Fig. S14). For further analysis, we utilized single exponential function fitting. Then normalized residuals were calculated as (observed value − fitted curve value)/(fitted curve value)^46^. These residuals were proposed as a measurement of the fractional MFE^46^. It is worth noting here that residuals, generally, are used to detect various types of disagreement between data and the assumed model^79^. Basically, residual analysis shows the quality of regression. The residuals randomly distributed around zero highlight the validity of a particular selected regression model for a given data set^79^. Contrary, fluctuating patterns of residuals around zero over time would suggest that the error term is variable^79^. In other words, it is a prerequisite of the uncertainty in the model, that suggests a lack of perfect goodness of a fit^79^.

Indeed, calculated normalized residuals for 90–107 cells showed a random distribution (Figs. S15 and S16). We were unable to observe any consistent patterned changes in the residuals corresponding to the magnetic field frequency or amplitude (Figs. S15 and S16). Corresponding averaged values of normalized residuals only support absence of any pattern associated with the MFE (Fig. 7B, Figs. S15 and S16). Interestingly, we could sub-select a small number of cells in control (without any field exposure), which showed either fluctuating or non-fluctuating patterns of residuals around zero (Fig. S17). To clarify, some HeLa cells randomly without any magnetic field exposure exhibited clear fluctuating patterns of residuals (Fig. S17).

**Figure 8:**
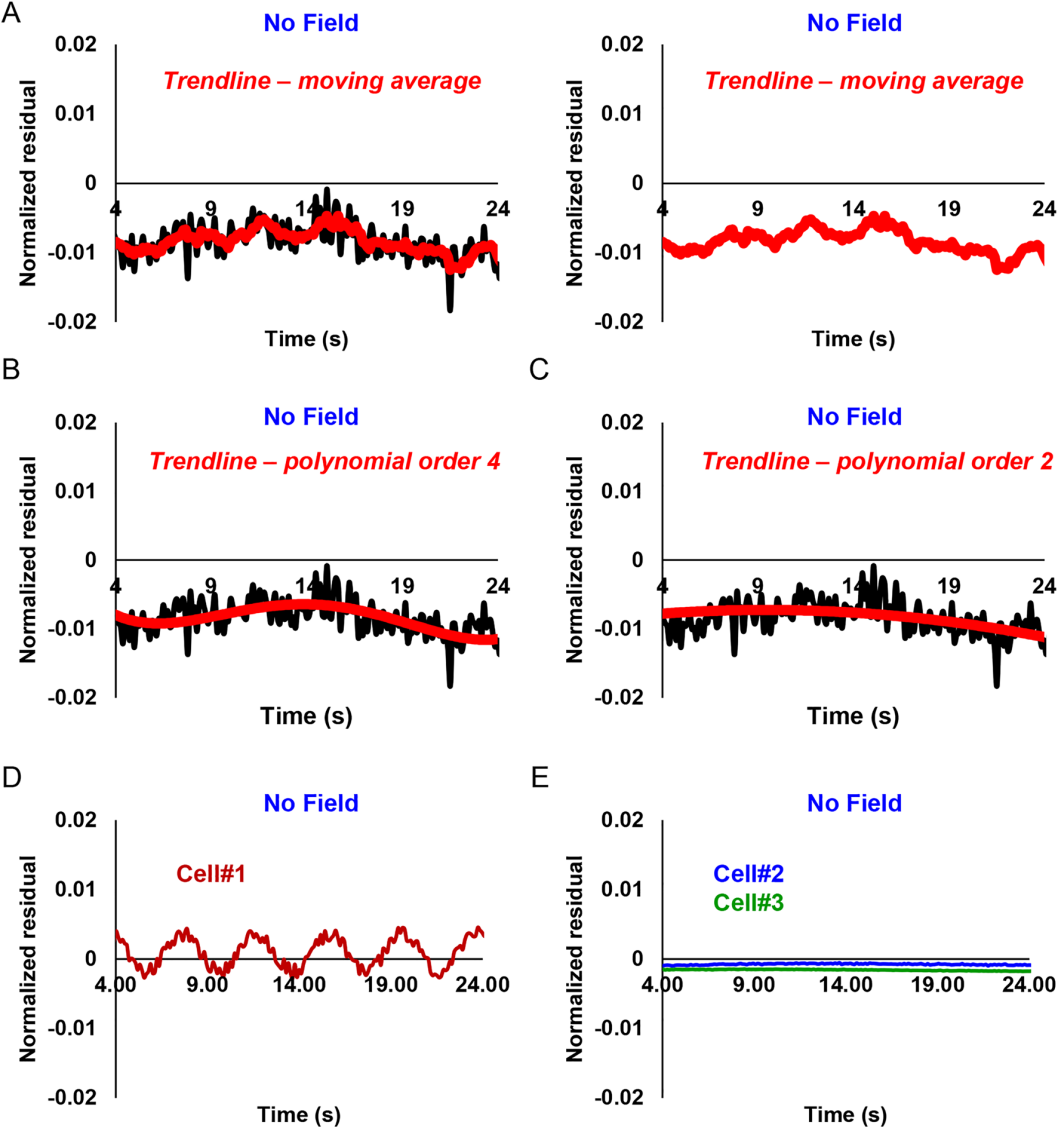
Fitting of normalized residuals taken form (Fig. 6C) of control cells not exposed to magnetic fields. Fitting of normalized residuals using moving average trendline (**A**), polynomial 4th order trendline (**B**), polynomial 2nd order trendline (**C**). (**D**,**E**) Selected single cell normalized residuals representing variability of observed patterns in control cells not subjected to magnetic fields. All graphs were created with Excel 365.

To underline our findings and stress random distribution of normalized residuals, we zoomed in the Y-axis to make small differences more visible (Fig. 7C). We were unable to identify any fluctuations of normalized residuals corresponding to any frequency of the applied modulated magnetic field (Fig. 7C). The residuals fluctuate in the same range as in^46^ giving only fraction of 2% change (Fig. 7C). Of note, sample size is a crucial factor in determining the power of a study and its reliability^71,72,80^. Detecting small differences between treatment and control groups requires especially large sampling^71,72,80^. This is absolutely required to avoid false positive findings due to human inherited ability to apophenia (the “tendency to perceive meaning in noise”)^81^. We can illustrate this ability by taking control distribution of normalized residuals and fitting this data to different mathematical models (Fig. 8A–C). One can observe different tendencies in the same data when different models are applied (Fig. 8A–C). Therefore, a large sample size and direct comparison control vs treatment are crucial in this case. Some individual cells without application of any magnetic field show oscillating pattern of normalized residuals (Fig. 8D). The other individual cells without application of any magnetic field do not have any patterns (Fig. 8E). However, with large sample size normalized residuals for 90–107 cells possess a random distribution (Fig. 7C, Figs. S15 and S16).

**Figure 9:**
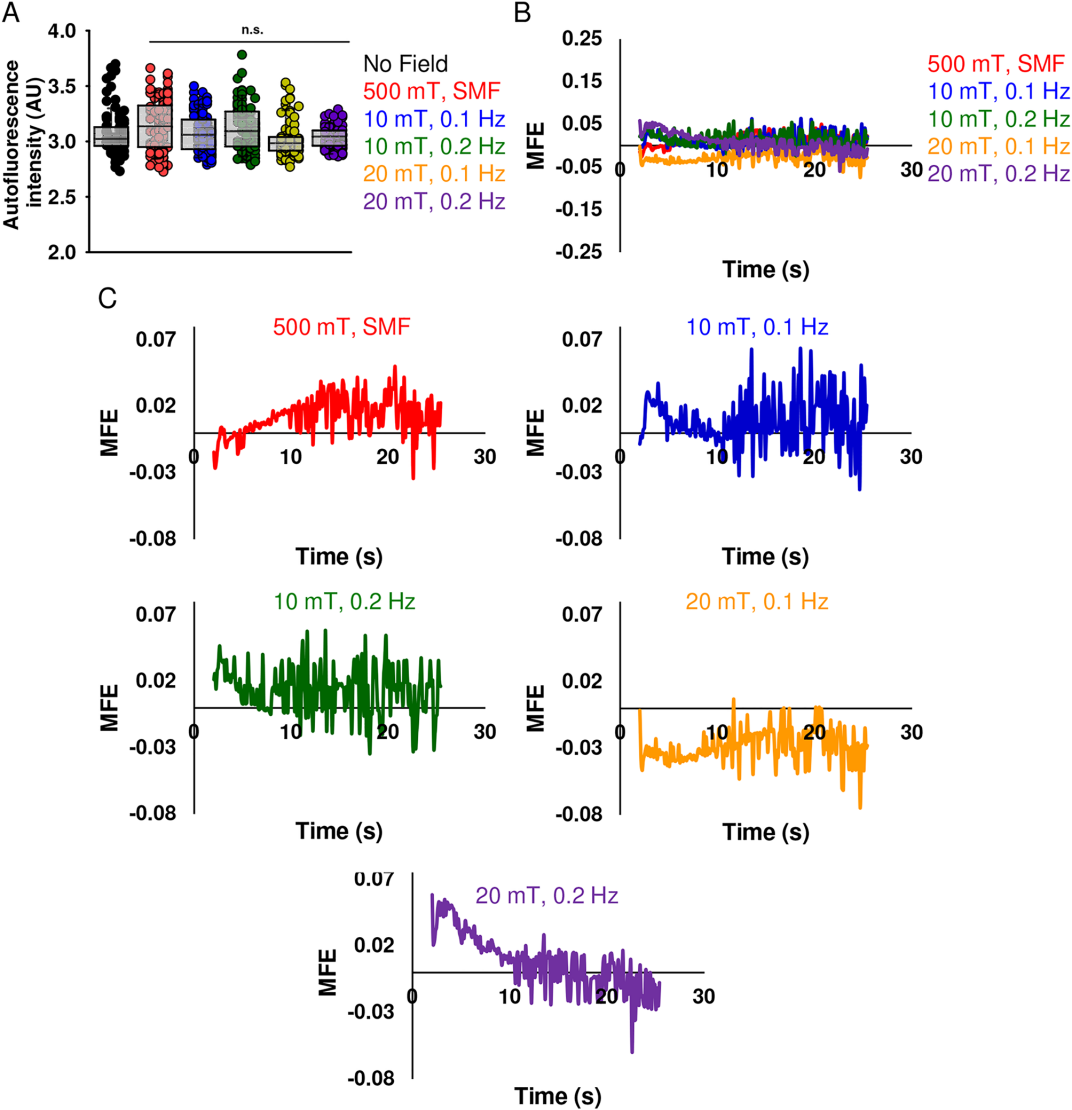
Magnetic field effect on HeLa cells autofluorescence intensity (**A**) Autofluorescence intensity comparison measured 20 s after exposure to different magnetic fields. Cells were irradiated by 10 mT and 20 mT modulated magnetic field (frequencies 0.1 Hz and 0.2 Hz). 500 mT static magnetic field (SMF) was generated by bulk NdFeB magnet. N = 90–107. Dunnett’s test was used to determine statistical significance. Differences were considered statistically significant at *P < 0.05 with respect to control (untreated cells). Graph was created using SigmaPlot 13.0. (**B**) Magnetic field effect (MFE) in the presence of static and a modulated magnetic field for averaged autofluorescence data calculated from Fig. 3A. (**C**) Magnetic field effect (MFE) curves from (**B**) presented as separated graphs with zoomed in Y-axis. Graphs were created with Excel 365.

We think that calculation of residuals does not allow one to make reasonable analysis/conclusion whether magnetic field affects cellular autofluorescence. Therefore, apart from large sample size it is important as well to directly compare control vs treatment measurements. It is important and widely accepted practice and accepted that a conclusion about an observed effect of a treatment should be based on a direct statistical comparison between a control and a treatment group^71^. We directly compared levels of cell autofluorescence at the 20th second after photobleaching in the presence and absence of magnetic fields (Fig. 9A and Fig. S18). We analyzed levels of cell autofluorescence of 90–105 individual cells. As can be seen, we observed no statistically significant difference in the autofluorescence between the control cells (no magnetic field group) and the cells exposed to any of the magnetic field conditions used (Fig. 9A).

It is worth noting that in a previous study dealing with MFEs on chemical reactions, MFE was defined as *[I*(*B*_*0*_)* − I*(*0*)*]/I*(*0*)^34,38^ or [*I*(*0*) *−* *I*(*B*_*0*_)*]*/*I*(*0*)^37^. *I*(*B*_*0*_) and *I*(*0*) are the fluorescence/absorption intensities in the presence and in the absence of the magnetic field respectively^34,37,38^. We performed further calculations of MFE, defined as [*I*(*B*_*0*_)* − I*(*0*)]/*I*(*0*). We found no trend in those data (Fig. 9B). Neither frequency nor amplitude dependence of MFE were observed in HeLa cells upon magnetic field exposure (Fig. 9B). Even if we zoom in Y-axis and present each treatment as separate curves for different treatments, there is no dependency of MFE corresponding to the frequency of the applied modulated magnetic (Fig. 9C). The data show random noisy distribution (Fig. 9C).

**Figure 10:**
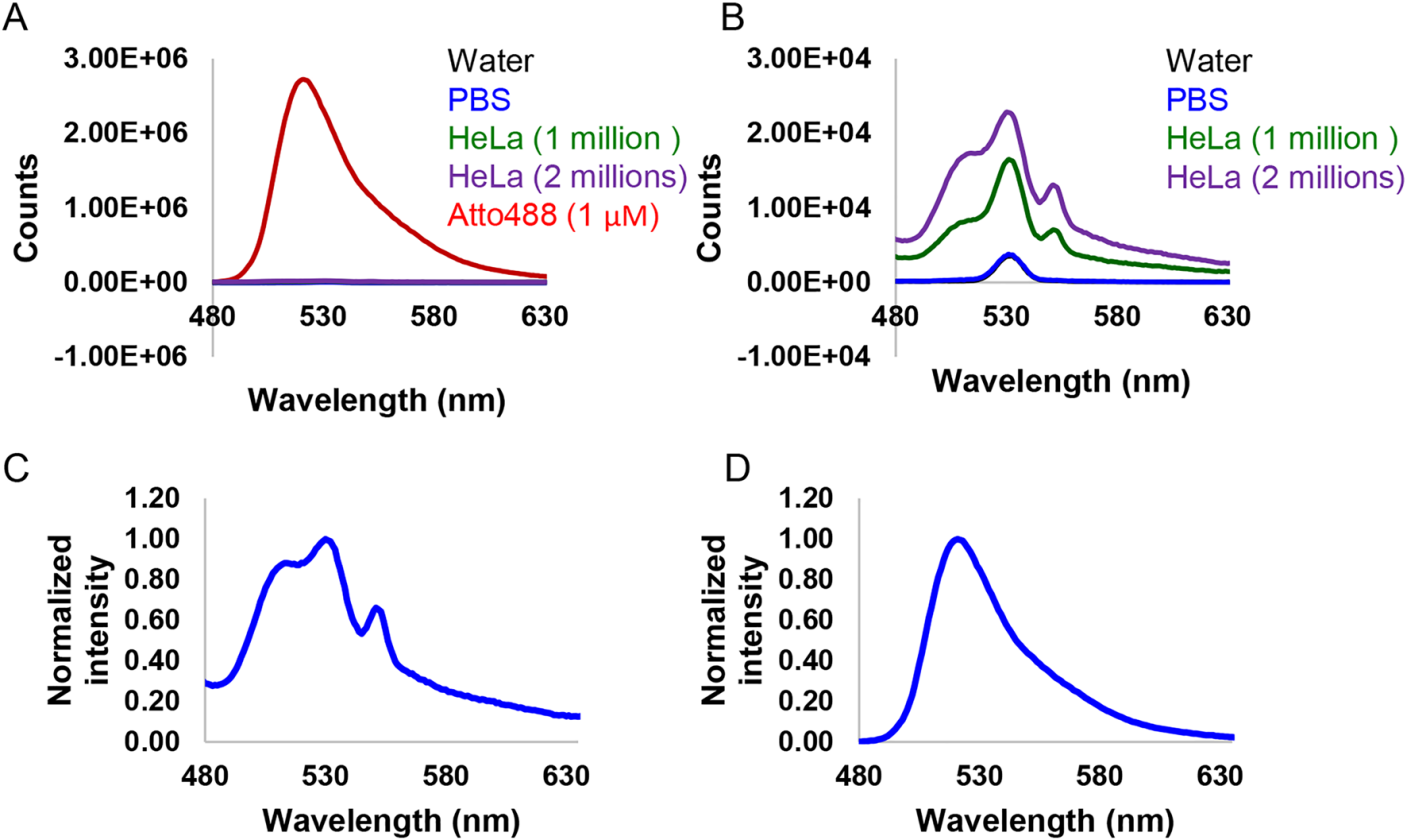
Fluorescence spectra of HeLa cells under 450 nm excitation. (**A**) Fluorescence spectra of water, PBS, HeLa cells diluted in PBS and Atto488 (1 μM in PBS). (**B**) Enlarged fluorescence spectra of water, PBS, HeLa cells diluted in PBS. (**C**) Normalized to the maximum intensity and adjusted for the Raman scattering fluorescence spectrum of HeLa cells diluted in PBS. (**D**) Normalized to the maximum intensity fluorescence spectrum of Atto488 (1 μM in PBS). Graphs were created with Excel 365.

In order to stress that autofluorescent signal from cells is weak, we measured fluorescence spectra in HeLa cells (Fig. 10). One can see that fluorescent signal from cells is orders of magnitude lower in comparison to our standard Atto488 dye (Fig. 10A). Moreover, the spectrum of cells had significant impact from Raman scattering of water (Fig. 10B). Generally, fluorescence can be more intense than the weak Raman scatter, and Raman interference is not presenting an issue for fluorescence spectra measurements. In fact, in diluted solutions of fluorophores the Raman scatter from the solvent can significantly distort the measured fluorescence spectrum^82^. This is what we have observed with HeLa cells (Fig. 10B). Interestingly, even after the solvent spectrum subtraction and normalization the HeLa cells spectrum had multiple peaks (Fig. 10C), not as smooth as normalized Atto488 spectrum (Fig. 10D). These findings only support our conclusions that autofluorescence of HeLa cells is weak and supposedly comprised of distinct chemical entities (e.g., FAD, FMN, lipofuscin, glycation adducts see Table S1).

## Discussion

Direct identification of biological effects of magnetic fields at the cellular level still remains an interesting and challenging task^1–4^. Reproducibility of key findings is a major challenge to this field of research^1–4,6,7,9,10^. When systematic or critical reviewing is applied to analyze inconsistencies in reports of the biological effects of magnetic fields, it quite often leads to the conclusion, that there are either insufficient descriptions of design, execution, or validation of the experimental methods and systems^2,4,6,7,10^. Detailed reporting of the key elements of experimental setup, as well as, mathematical and statistical analysis of the generated data are crucial for the effective reproducibility of the results and establishing findings as scientifically verified facts^83^. Deviation from these guidelines may lead to problems and the claimed findings may only represent measures of the prevailing bias^84^. Due to the lack of reproducibility of the results on biological effects of magnetic fields at the cellular level^11–15^, it is important to verify independently claims about MFEs. The multidisciplinary area of biological effects of magnetic fields suffers from dramatic variability in experimental details reported, usage and characterization of biological models. This, in turn, creates a significant barrier to progress research forward. Therefore, we suggest that research community should establish a ‘minimum information standard’ for research dealing with biological effects of magnetic fields, which already exist for biochemical models^85^, genome sequencing^86^, quantitative PCR^87^, animal research^88^, and bio–nano experimental literature^89^.

In this study, we investigated the magnetic field sensitivity of an endogenous autofluorescence in HeLa cells. We would like to stress here, that we do not claim that magnetic field effects in general or alteration of biochemical reactions by magnetic field via so-called radical pair mechanism do not exist. Of note, there is evidence for external magnetic fields being capable of affecting different chemical reactions kinetics in artificial systems in vitro, for review see^3,36^ and references therein. Studies show that magnetic fields can perturb radical pair reactions in artificial cell-free systems of flavin/tryptophan molecules^34,37–41^. However, biochemical reactions observed in dilute buffer systems most often do not represent those in the cellular environment^42^. Currently, living cells are not recognized anymore as a “bag of enzymes”^43^. High internal concentration of macromolecules, the constraints of cellular architecture (confinement is created by cytoskeletal elements, membrane structures) greatly affects the equilibria, rates of biochemical reactions and diffusion of molecules^42–45^. As a result, biological fluids, in general, appear to be more complex than diluted artificial buffer systems studied theoretically or experimentally in vitro^42^. Therefore, it is crucial to validate results obtained in cell-free systems using real cells to account for increased complexity and heterogeneity of the system.

Based on cellular autofluorescence decay measurements, we show that this is not the case and the observed autofluorescence is not magnetic field dependent under the experimental conditions used in this study. In fact, there are many endogenous compounds responsible for 500–550 nm range of autofluorescence (Table S1). Flavins are only a part of the cocktail of compounds contributing to cellular “green” autofluorescence (Table S1). We demonstrate that “green” autofluorescence is highlighted by at least two (i.e., tubular, and vesicular) distinct subcellular (compartments) structures (Fig. 2B and Fig. S4). Our findings are in line with literature that identifies the mitochondria and lysosomes as the main subcellular structures that contribute to endogenous autofluorescence^68,69^. Of note, lysosomal autofluorescence is a result of lipofuscin accumulation^68,69^. Moreover, we performed colocalization analysis by labeling mitochondria and lysosomes (Fig. 3). This analysis confirmed that autofluorescent signal from single cell originates from both mitochondria and lysosomes simultaneously (Fig. 3). Thus, one should consider the contribution of lipofuscin in cellular autofluorescence signal.

We found following features of the cellular autofluorescence signal. Levels of autofluorescence intensity vary between different cells (Fig. S6). Autofluorescent structures can move at relatively high speed (Fig. 2C and Movie S3). All these factors contribute to the large variability in the observed autofluorescence decay upon photobleaching (Fig. S7 and Movies S4 and S5), and this observed variability may spill over into the quantitative analysis of images^53,67^. Indeed, this is not surprising, because the level of cellular autofluorescence depends on multiple factors, e.g. metabolic activity^90^, cell cycle phase^91^, cell aging and level of oxidative stress^92^, degree of cell damage and cell death^93,94^. Given complexity of endogenous sources of autofluorescence and multiple factors affecting its levels, it is of no surprise, that the origin of autofluorescence photobleaching is still not completely understood^95^. As a result, several distinct decay models can be used for fitting the photobleaching dynamics, ranging from one- to three exponential decay functions^95–97^. We have shown that photobleaching dynamics can be nicely fitted using two different models (Fig. S14).

It is worth noting that the calculation of the residuals as (observed value − fitted curve value)/(fitted curve value)^46^ can be an indicator of the goodness of the selected fitting model^79^. Direct comparison of control cells with cells exposed to a magnetic field is required to make a reasonable interpretation. It is nicely summarized in^71^, how omitting direct comparison can lead to misleading conclusions. This is exemplified in our findings that the application of a magnetic field of different amplitude and frequency did not result in any noticeable effect on the autofluorescence decays in HeLa cells (Fig. 7A and Fig. S13).

The magnitude of a magnetic response was reported from 1 to 2.5%^46^. With such small size effects one should be extremely cautious about interpretation and thus acquire large sampling numbers to validate the observation^71^. Utilization of small sample sizes precludes low reproducibility of results and potential skewing of the observed effect^72^. Cell cultures are heterogeneous, biochemically active, and thermodynamically open systems^98^. Compartmentalization, high internal concentration of various macromolecules and the constraints of cellular architecture can greatly influence intracellular biochemical reactions, making it sometimes very difficult to directly translate results from chemical system in test tube to a biological system (cell culture)^43^. Specifically, HeLa cells possess protein expression heterogeneity, the genomic and proteomic changes caused by successive passaging, and those variables greatly influence common cell assays^99^. The level of cellular autofluorescence is variable and is affected by many factors, e.g. metabolic activity^90^, cell cycle phase^91^, cell aging and level of oxidative stress^92^, degree of cell damage and cell death^93,94^. FAD concentration and subcellular localization undergo continuous changes in living cells^100^.

Importantly, while measuring an outcome at multiple time points, changes may arise due to other factors not relating to the treatment. Repeating the same measurement continuously may bring noticeable changes between pre- and post-intervention measurements originating from experimental bias and/or due to other changes relating to the passage of time but not treatment itself^71,101^. Therefore, for studies experimentally analyzing impact on a variable over time, it is necessary to directly compare the effect of experimental treatment with the effect of an unaffected control group^71,101^. This is needed to assure that the observed effect is larger than variability over time and that is not a product of experimental bias and/or error^71,101^.

When we acquired a large number of samples (N = 90–107), the patterned changes in the residuals corresponding to the magnetic field frequency or amplitude were not detected (Figs. S15 and S16). The averaged values of normalized residuals underline absence of the MFE on autofluorescence decay in HeLa cells (Fig. 7B,C, Figs. S15 and S16). Initial huge turbulence in the autofluorescence decay signal, multiple endogenous and exogenous factors affecting its levels, compounded with a lack of clarity around how the calculations of the fractional MFE were performed, all may have led to observations of fluctuating patterns of residuals around zero importantly in a very small number of cells (sampling), even in the absence of field exposure (Fig. S17).

In conclusion, we were not able to observe MFEs excreted on an endogenous autofluorescence in HeLa cells. Specifically, we found no noticeable magnetic field effect on autofluorescence decay course in HeLa cells. Collectively, we conclude that under the experimental conditions used in this study no MFEs have been observed in the autofluorescence of HeLa cells, due to (i) turbulent and weak autofluorescent signal from single cells, (ii) dependence of the autofluorescence on multiple factors, (iii) cellular autofluorescence representing a sum of signals contributing from distinct endogenous compounds.

## Supplementary Information

Supplementary Information 1.

Supplementary Video 1.

Supplementary Video 2.

Supplementary Video 3.

Supplementary Video 4.

Supplementary Video 5.

Supplementary Video 6.

Supplementary Information 2.

## Data Availability

The data generated and analyzed during this study are included in the body of the paper and the Supporting Information. Any additional datasets are available from the corresponding author on reasonable request.
