# Supplementary material for: Sensitivity of endogenous autofluorescence in HeLa cells to the application of external magnetic fields
Source: Sci Rep. 2023 Jul 4;13:10818. doi: 10.1038/s41598-023-38015-x (PMC10319795; doi:10.1038/s41598-023-38015-x)
Supplement: Supplementary file 8 — Supplementary Information 2. [file 41598_2023_38015_MOESM8_ESM.docx]

Supporting Information

Sensitivity of endogenous autofluorescence in HeLa cells to the application of external magnetic fields

Mariia Uzhytchak ^1^, Barbora Smolková ^1^, Adam Frtús ^1^, Alexandr Stupakov ^1^, Mariia Lunova ^1,2^, Federica Scollo ^3^, Martin Hof ^3^, Piotr Jurkiewicz ^3^, Gareth John Sullivan ^4,5,6^, Alexandr Dejneka ^1^, Oleg Lunov ^1,^*

*^1^ Department of Optical and Biophysical Systems, Institute of Physics of the Czech Academy of Sciences, Prague, 18221, Czech Republic*

*2 Institute for Clinical & Experimental Medicine (IKEM), Prague, 14021, Czech Republic*

*^3^ J. Heyrovský Institute of Physical Chemistry of the Czech Academy of Sciences, Prague, 18223, Czech Republic*

*^4^ Department of Molecular Medicine, Institute of Basic Medical Sciences, University of Oslo, Oslo, Norway*

*^5^ Department of Pediatric Research, Oslo University Hospital, Oslo, Norway*

*^6^ Department of Immunology, Institute of Clinical Medicine, University of Oslo, Oslo, Norway*

^*^ Corresponding author. Department of Optical and Biophysical Systems, Institute of Physics of the Czech Academy of Sciences, Prague, 18221*,* Czech Republic. Tel.: +420266052131.

*E-mail address: lunov@fzu.cz* (O. Lunov)

Number of pages: 21

Number of tables: 2

Number of figures: 18

Number of movies: 6

**Table of Contents**

Table S1 S3

Table S2 S3

Figure S1 S4

Figure S2 S4

Figure S3 S5

Figure S4 S5

Figure S5 S6

Figure S6 S7

Figure S7 S7

Figure S8 S8

Figure S9 S9

Figure S10 S10

Figure S11 S11

Figure S12 S12

Figure S13 S13

Figure S14 S14

Figure S15 S15

Figure S16 S16

Figure S17 S17

Figure S18 S18

Legends for Movies S19

References S20

Table S1. Selected endogenous fluorophores responsible for cell and tissue autofluorescence.

| Molecule | Excitation, peak position range (nm) | Fluorescence, peak position range (nm) | Ref. |
| --- | --- | --- | --- |
| Flavin adenine dinucleotide (FAD) | ~ 380-490 | ~ 520-560 | (1) |
| Flavin mononucleotide (FMN) | ~ 380-490 | ~ 520-560 | (1) |
| Lipofuscin | ~ 410-488 | ~ 500-695 | (2) |
| Elastin | ~ 350-420 | ~ 420-510 | (3) |
| Glycation adducts of collagen | ~ 370-420 | ~ 450–460 | (4) |
| Free fatty acids (arachidonic,  linoleic, linolenic acid) | ~ 330-350 | ~ 470-480 | (5) |
| Biliary salts and bilirubin | ~ 400-490 | ~ 540-600 | (5) |

Table S2. Quantum yield and brightness of different compounds.

| Molecule | Quantum yield | Molar extinction coefficient (M^-1^ cm^-1^) | Brightness^1^ | Ref. |
| --- | --- | --- | --- | --- |
| Riboflavin | 0.36 | 33000 | 11880 | (6, 7) |
| Flavin mononucleotide (FMN) | 0.26 | 12200 | 3172 | (8, 9) |
| Flavin adenine dinucleotide (FAD) | 0.033 | 11300 | 372.9 | (10, 11) |
| A2E (one of the fluorophores in lipofuscin) | 0.01 | 36900 | 369 | (12, 13) |
| Tryptophan | 0.12 | 5540 | 664.8 | (14, 15) |
| Fluorescein (FITC) | 0.95 | 76000 | 72200 | (16, 17) |
| Alexa Fluor 488 | 0.92 | 73000 | 67160 | (18) |

^1^The brightness is a product of extinction coefficient and quantum yield of the fluorophore (19).


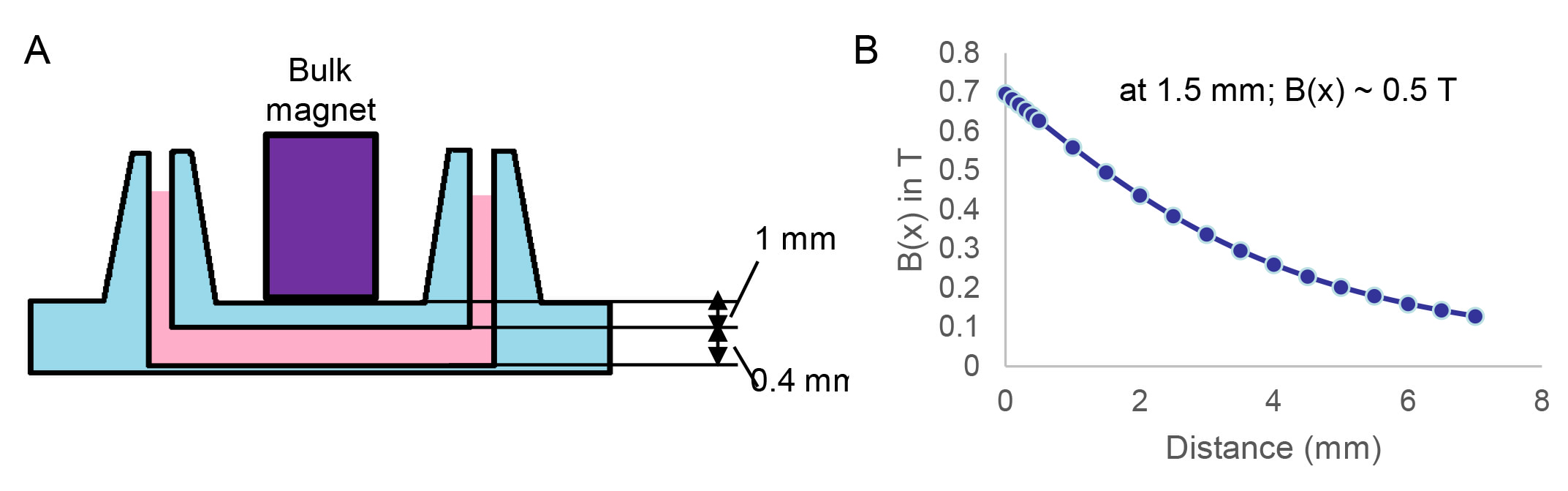


Figure S1. Estimation of the magnetic field generated by bulk NdFeB magnet. (*A*) Schematic application of bulk NdFeB magnet. (*B*) Calculation of distance decay of the flux density B(x), utilizing approached described in (20).

**Figure S2.** Measurements of magnetic environmental noise. (*A*) Background electromagnetic noise measurements. (*B*) Fourier power spectrum of the background electromagnetic noise.


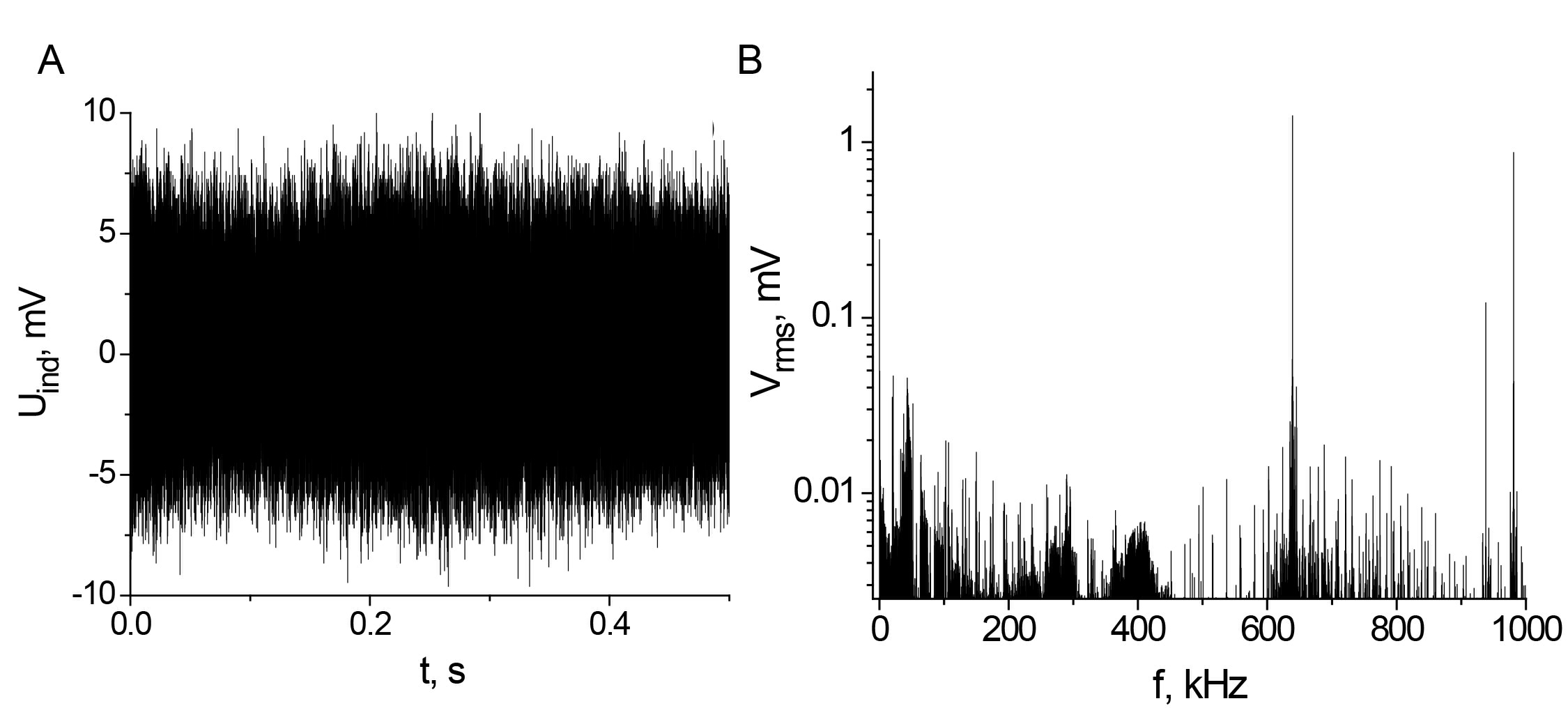

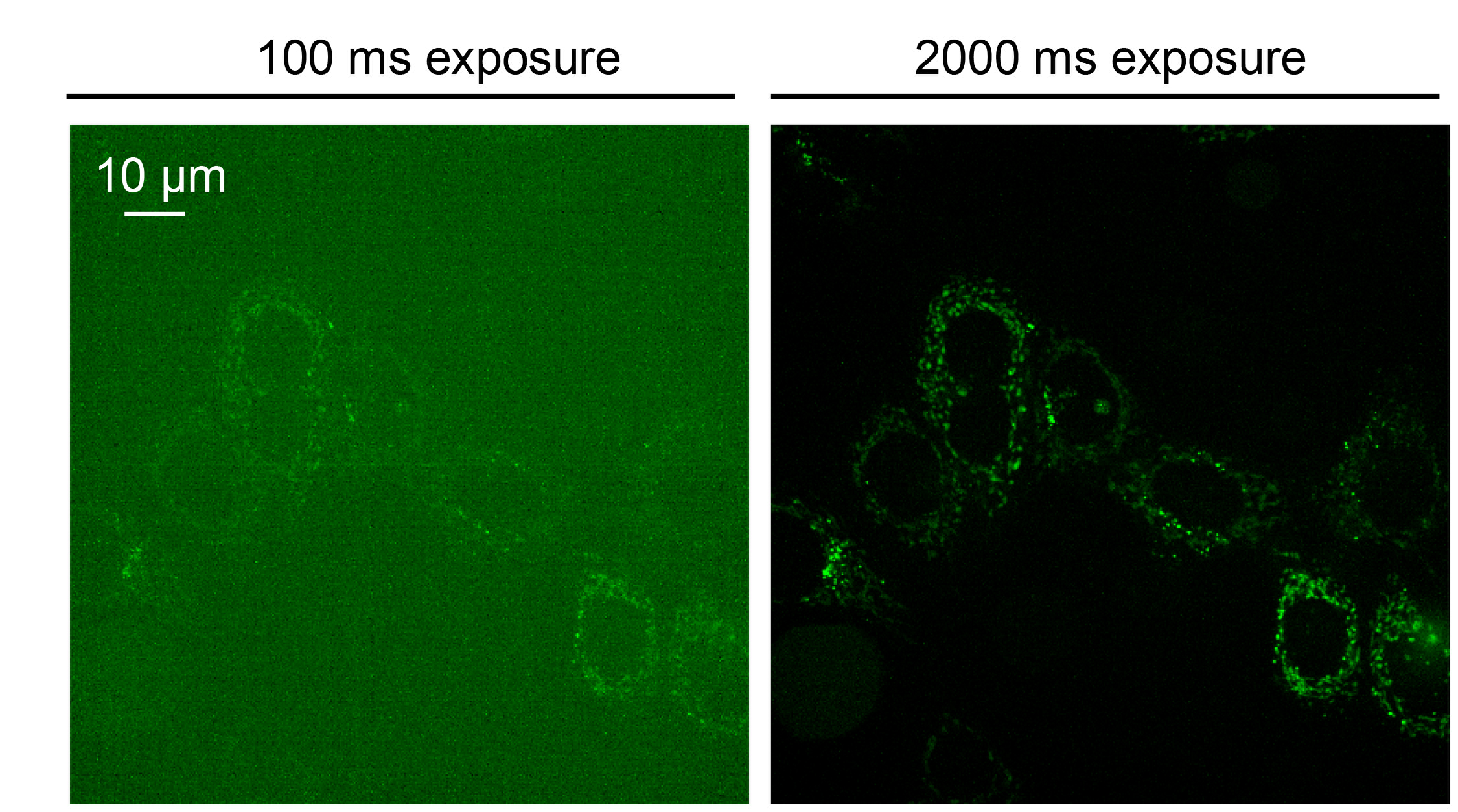


Figure S3. Full view of HeLa cells autofluorescence confocal images. Autoflourescence images of HeLa cells taken with 100 ms and 2000 ms exposure time.


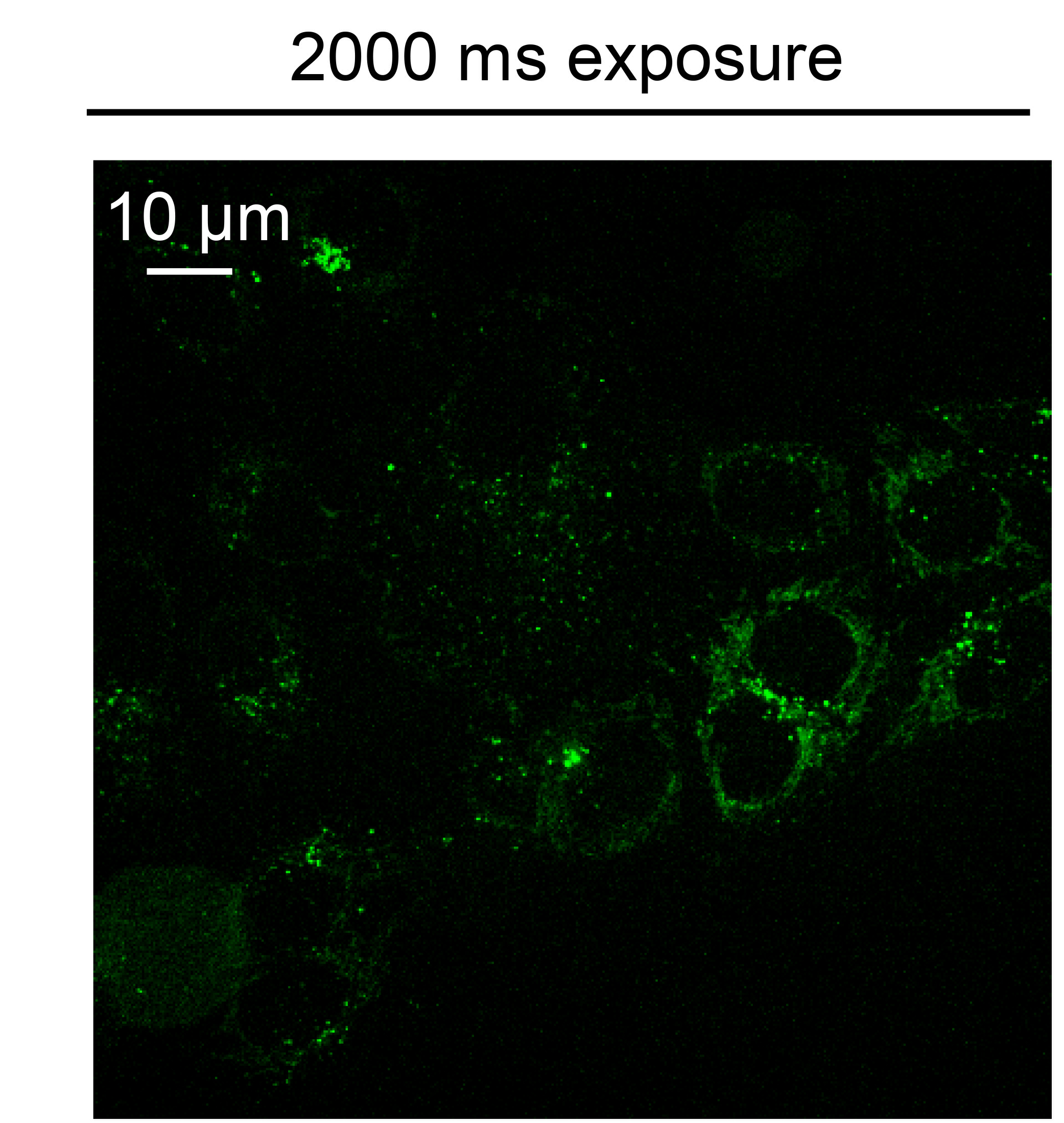


Figure S4. Full view image representing tubular and round intracellular structures in HeLa cells taken with 2000 ms exposure time.


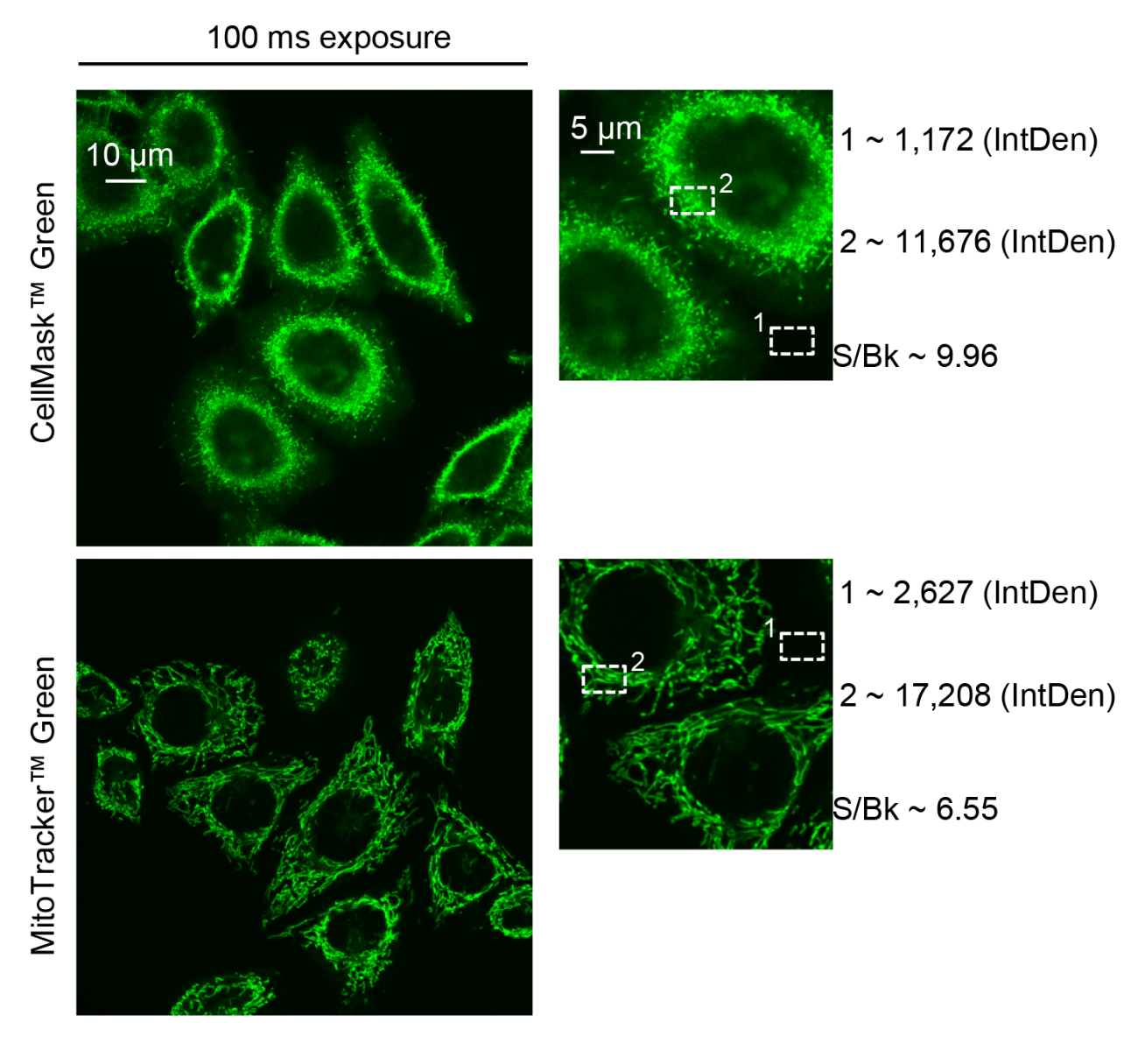


Figure S5. Fluorescent intensity measurements of HeLa cells stained with standard probes. Cells were labeled with CellMask Green (Thermo Fisher Scientific, US) in order to visualize plasma membrane. MitoTracker Green FM (0.5 μM), (Thermo Fisher Scientific, US) was used for mitochondria staining. Signal-to-background ratio (S/Bk) is calculated for selected image regions. Integrated density (*IntDen*) was measured for the background (1) and cell part (2) using ImageJ software (NIH, US).


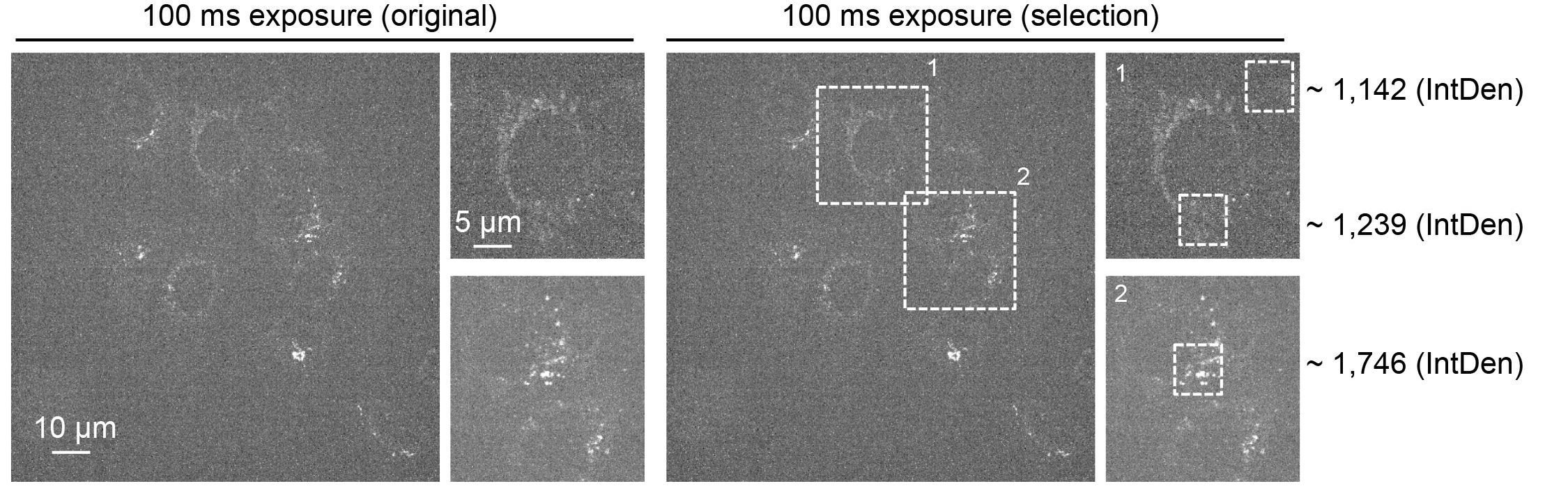


Figure S6. Cell autofluorescence presented in grayscale images with 100 ms exposure time. Integrated density (*IntDen*) was measured for the background and cells regions using ImageJ software (NIH, US).


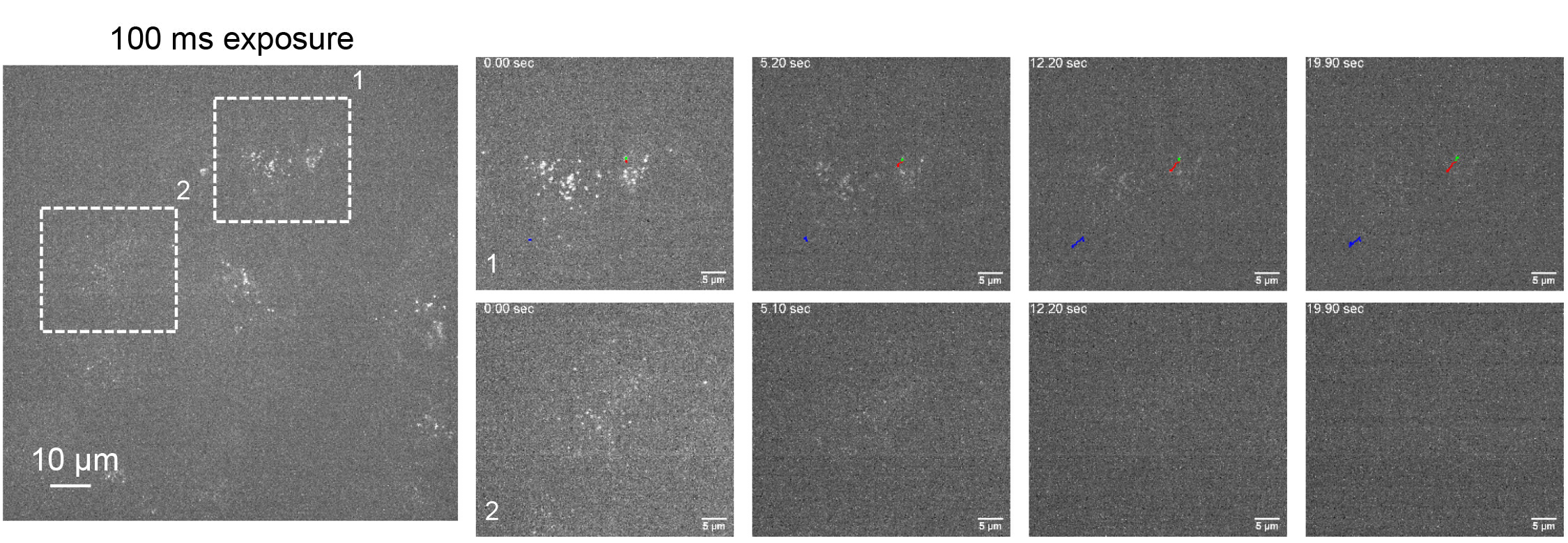


Figure S7. Autofluorescence decay time-lapse of HeLa cells within 20 sec period of laser irradiation. Cell autoflourescence decay presented in grayscale images with 100 ms exposure. Blue, red and green colors represent vesicle movements through the time-lapse image (Movies S4, S5 and S6).


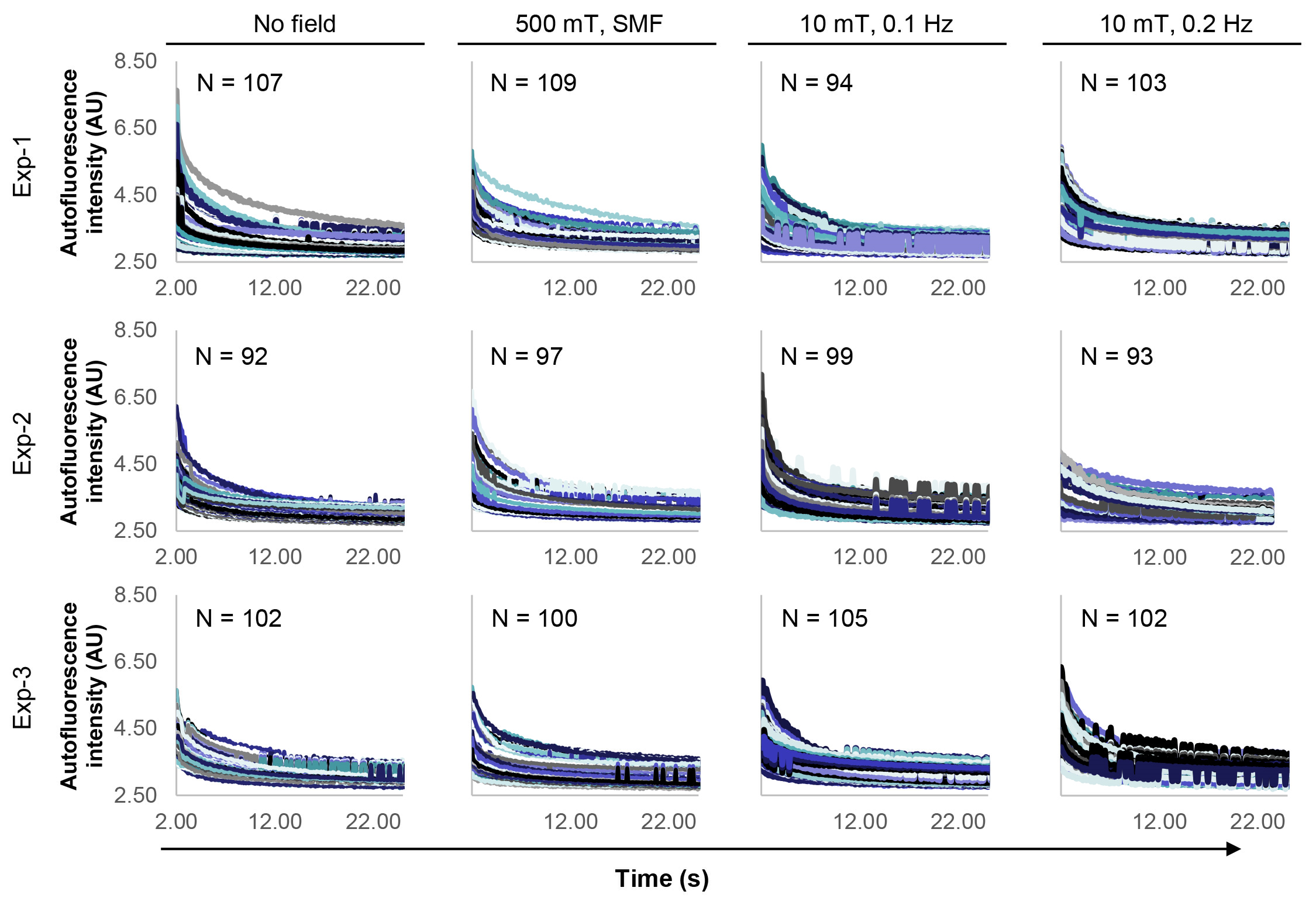


Figure S8. Original autofluorescence decay of HeLa cells. Cells were irradiated by 10 mT modulated magnetic field (frequencies 0.1 Hz and 0.2 Hz). 500 mT static magnetic field (SMF) was generated by bulk NdFeB magnet.


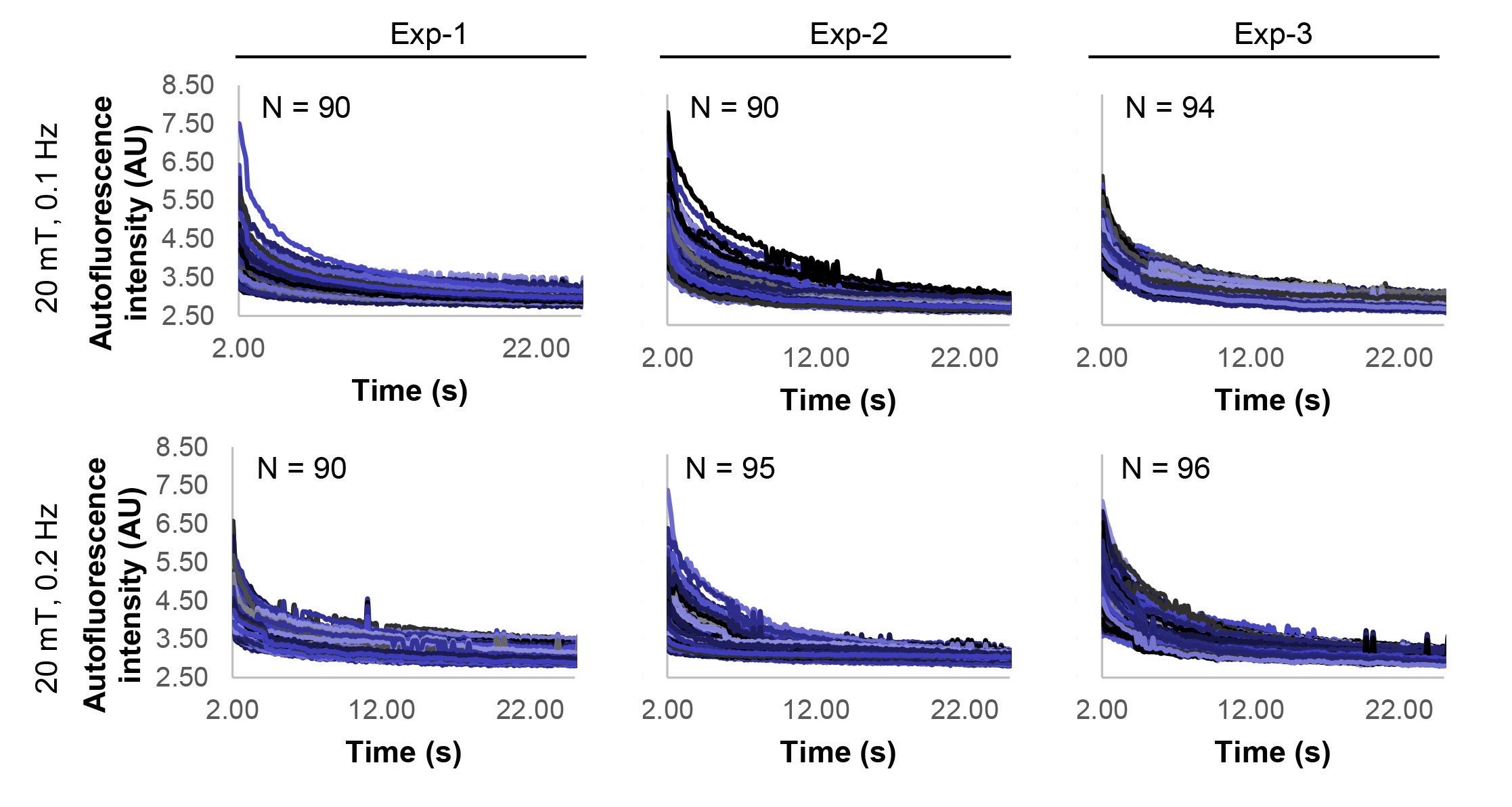


Figure S9. Original autofluorescence decay of HeLa cells. Cells were irradiated by 20 mT modulated magnetic field (frequencies 0.1 Hz and 0.2 Hz).


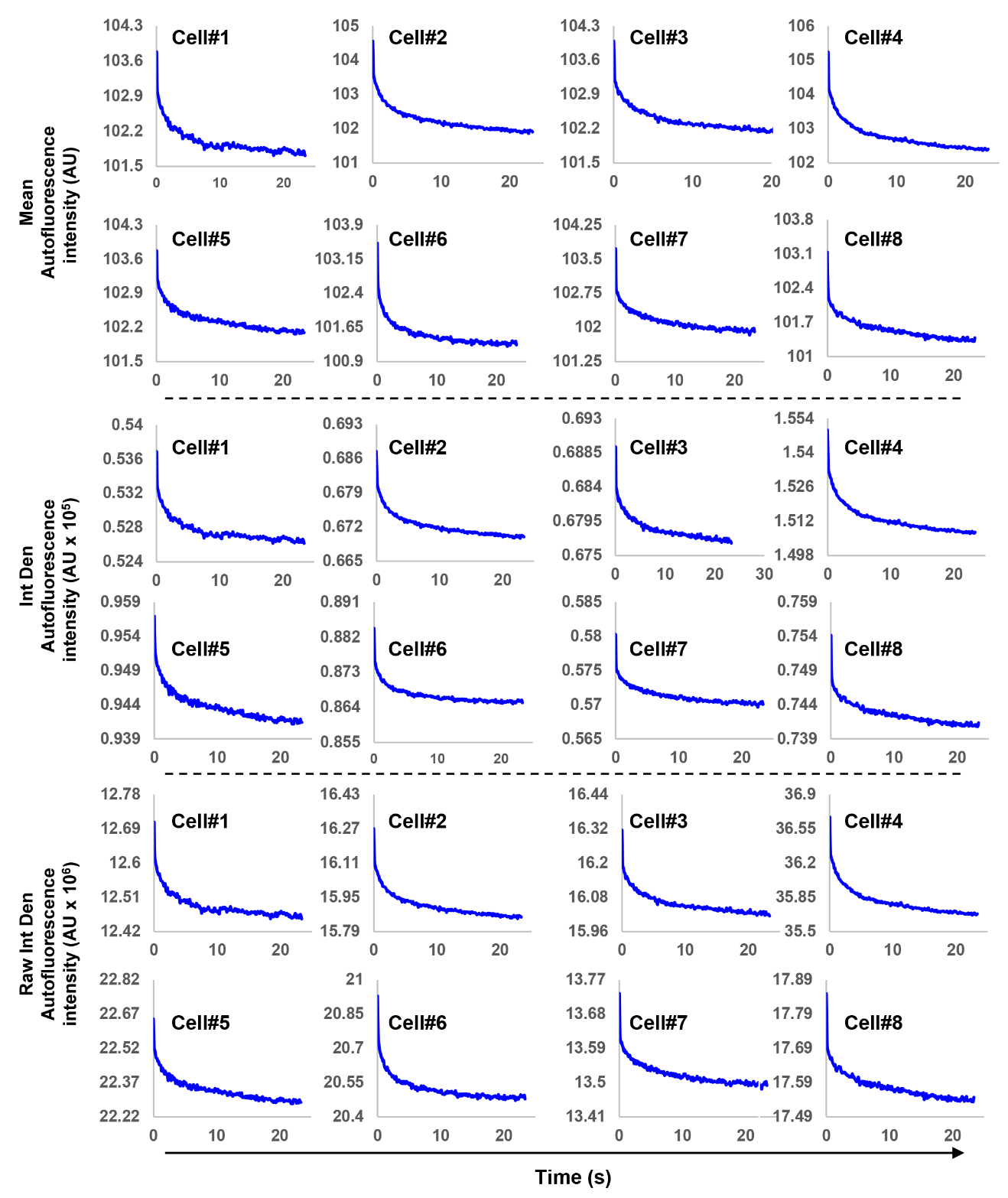


Figure S10. Averaged autoflourescence decay of control (no magnetic field exposure) cells. Single cell averaged autoflourescence decay of cells presented as mean gray value (Mean), integrated density (Int Den), and raw integrated density (Raw Int Den). Autoflourescence decay of cells as assessed by ImageJ software (NIH).


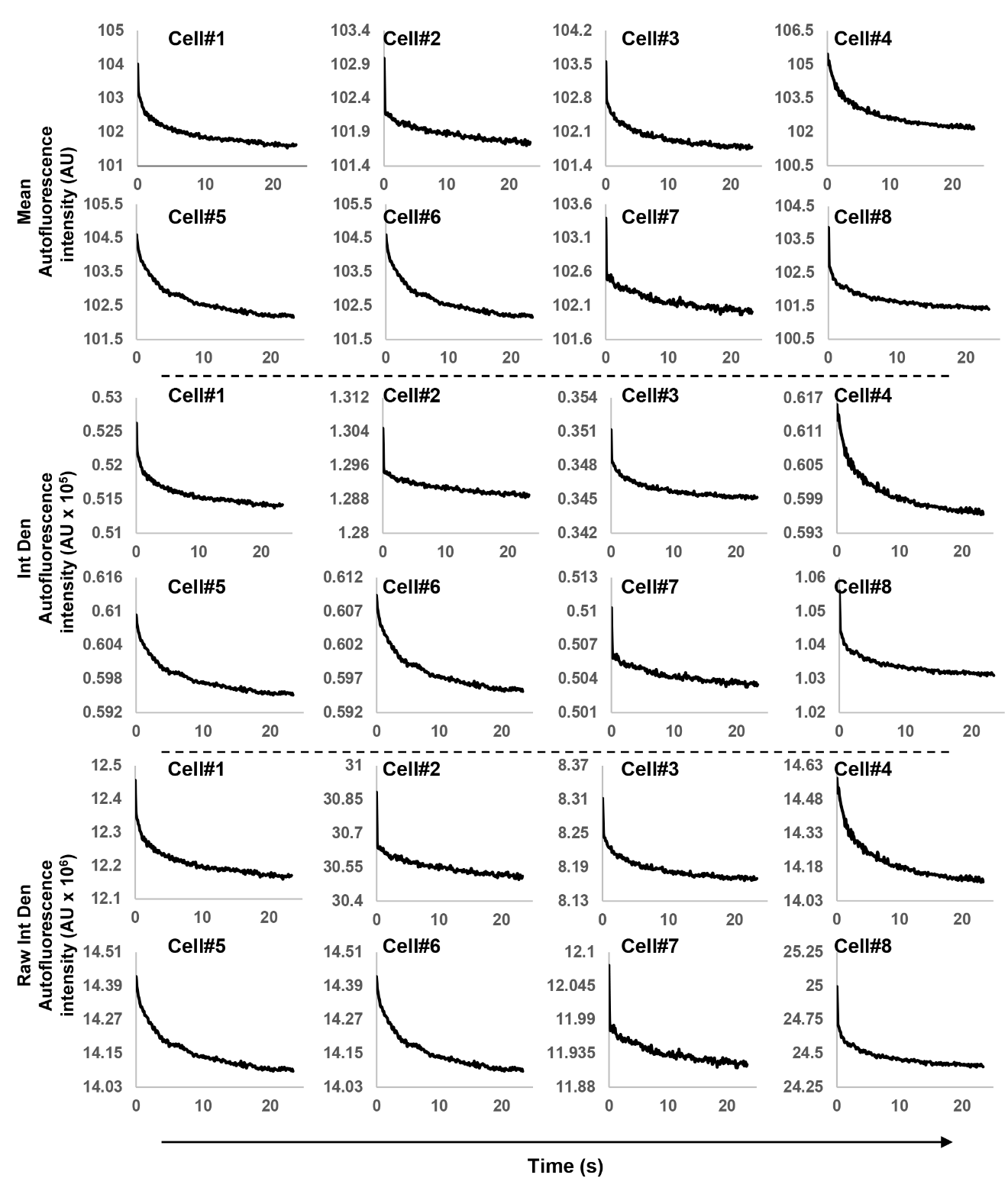


Figure S11. Averaged autoflourescence decay of cells exposed to a modulated magnetic field 10 mT (frequency 0.1 Hz). Single cell averaged autoflourescence decay of cells presented as mean gray value (Mean), integrated density (Int Den), and raw integrated density (Raw Int Den). Autoflourescence decay of cells as assessed by ImageJ software (NIH).


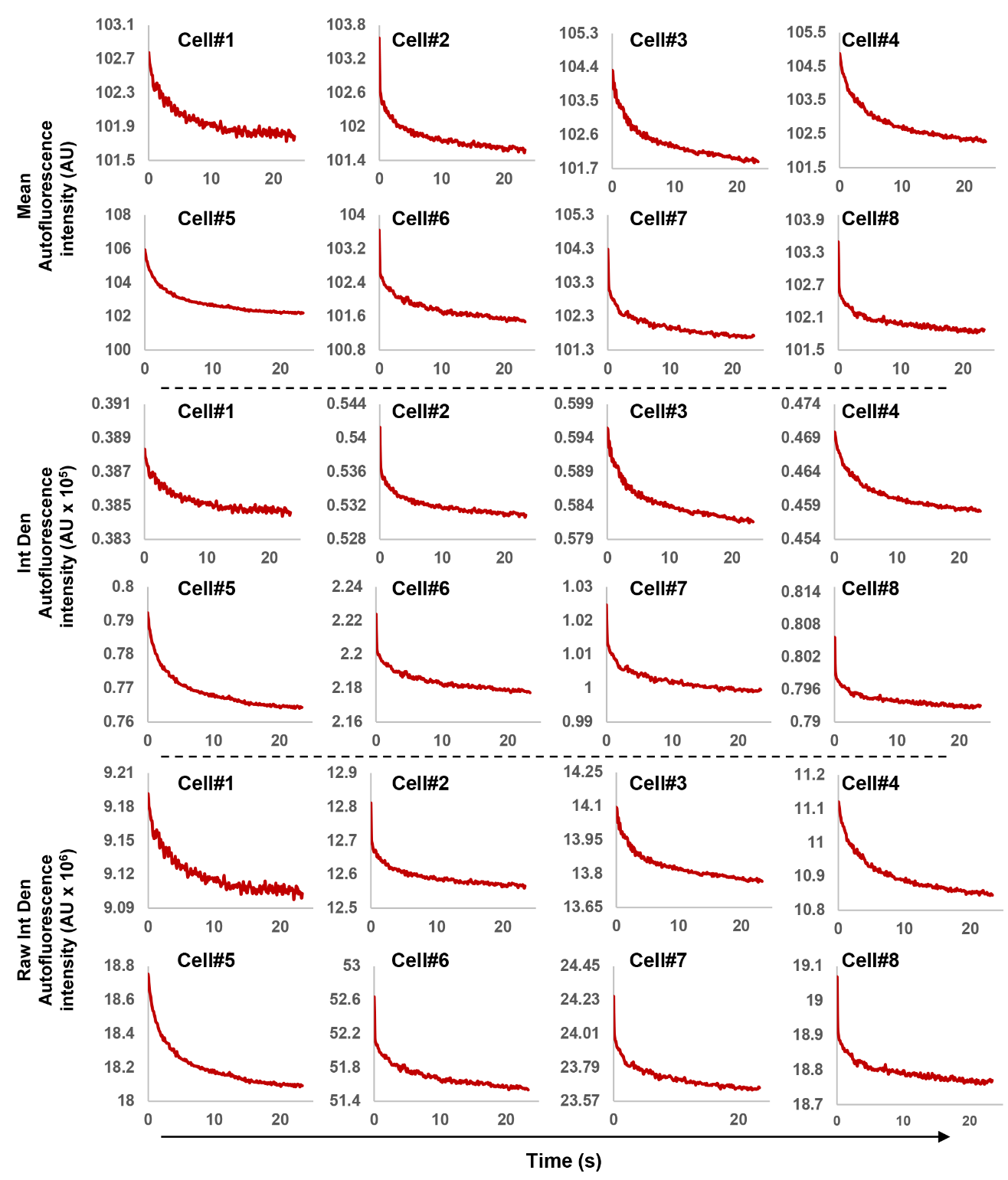


Figure S12. Averaged autoflourescence decay of cells exposed to a modulated magnetic field 10 mT (frequency 0.2 Hz). Single cell averaged autoflourescence decay of cells presented as mean gray value (Mean), integrated density (Int Den), and raw integrated density (Raw Int Den). Autoflourescence decay of cells as assessed by ImageJ software (NIH).


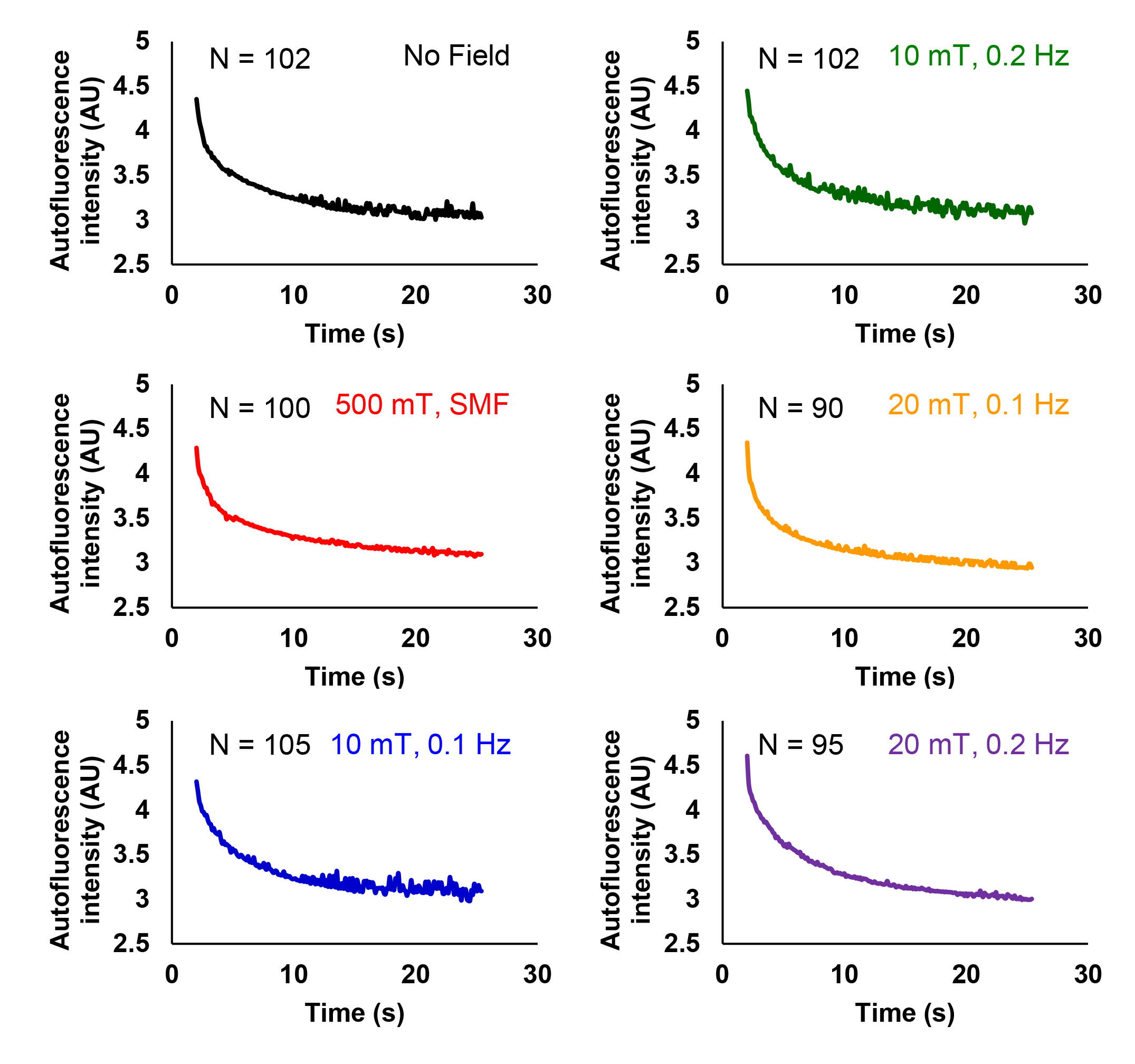


Figure S13. Average autofluorescence decay upon different magnetic field exposure. Cells were irradiated by 10 mT or 20 mT modulated magnetic field (frequencies 0.1 Hz and 0.2 Hz). 500 mT static magnetic field (SMF) was generated by bulk NdFeB magnet.


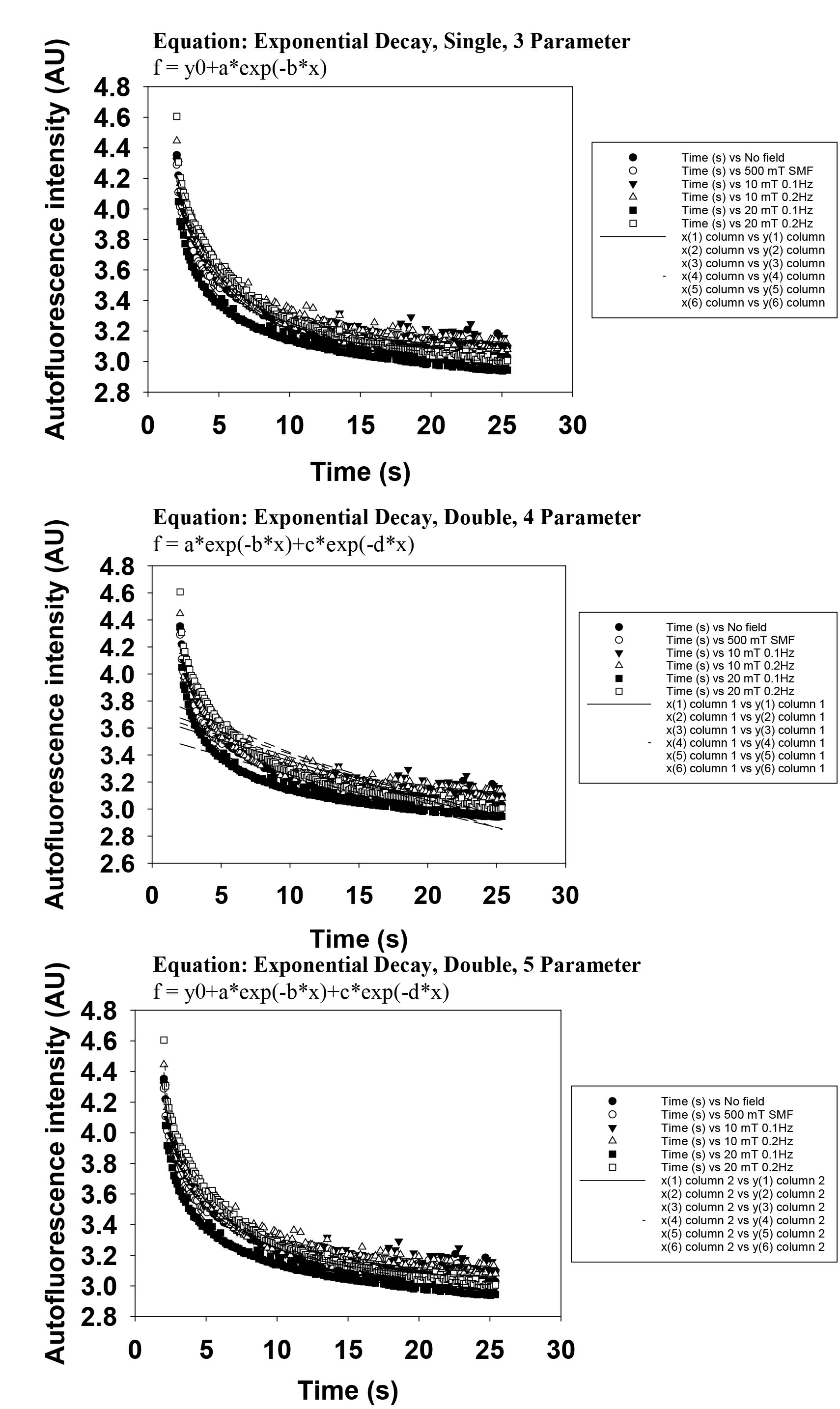


Figure S14. Fitting of average autofluorescent intensity decay using different functions, e.g. single exponential decay, 3 parameter; double exponential decay (Goodness of fit R = 0.9815; Rsqr = 0.9633, Adj Rsqr = 0.9627, SEE = 0.0486), 4 parameter; double exponential decay (Goodness of fit R = 0.8506; Rsqr = 0.7236, Adj Rsqr = 0.7194, SEE = 0.1305), 5 parameter (Goodness of fit R = 0.9893; Rsqr = 0.9788, Adj Rsqr = 0.9783, SEE = 0.0371). Displayed data were analyzed in SigmaPlot 13.0 software (Systat Software Inc., US).


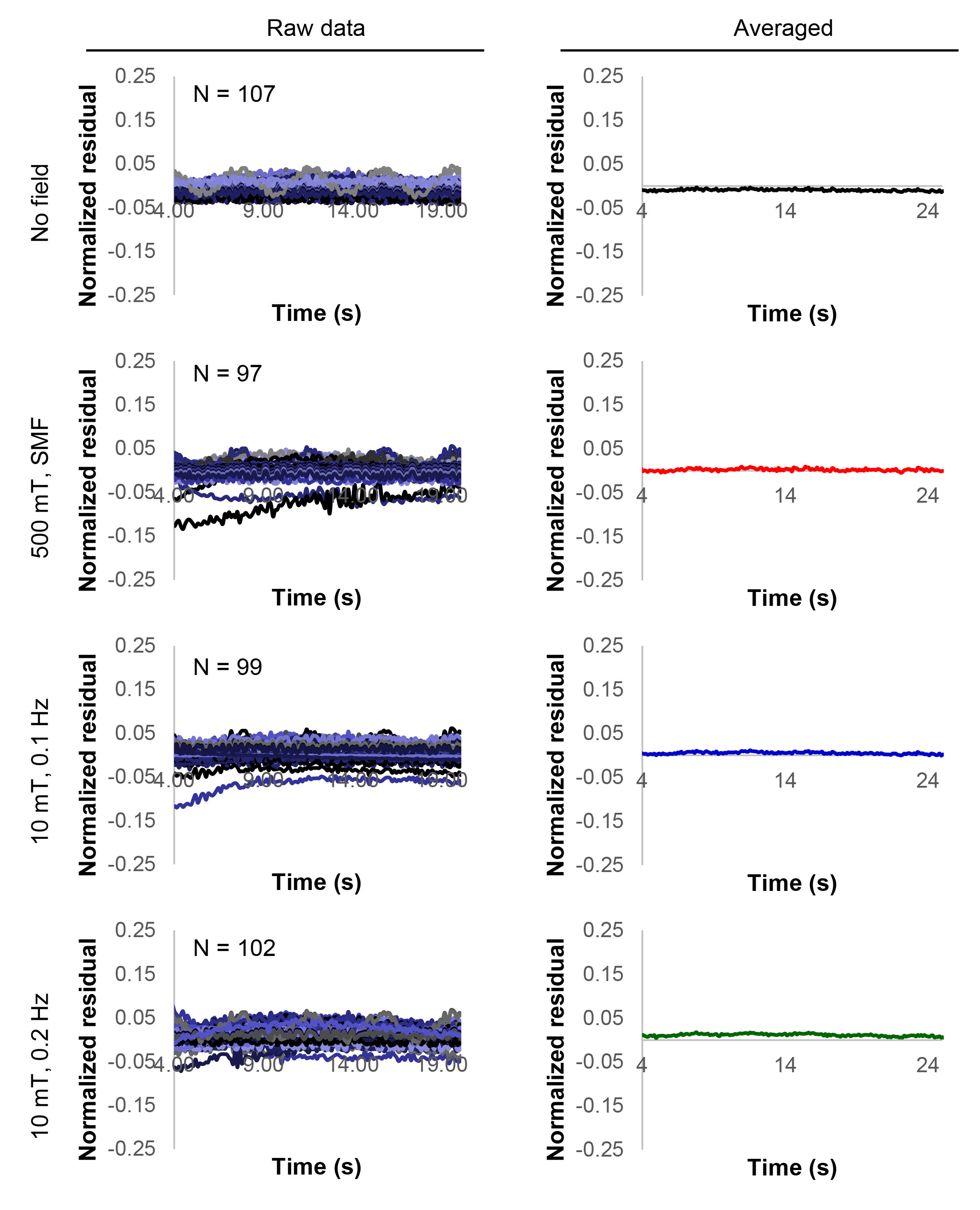


Figure S15. Raw data of normalized residuals of autoflourescent intensity response and corresponding averages. Cells were irradiated by 10 mT modulated magnetic field (frequencies 0.1 Hz and 0.2 Hz). 500 mT static magnetic field (SMF) was generated by bulk NdFeB magnet.


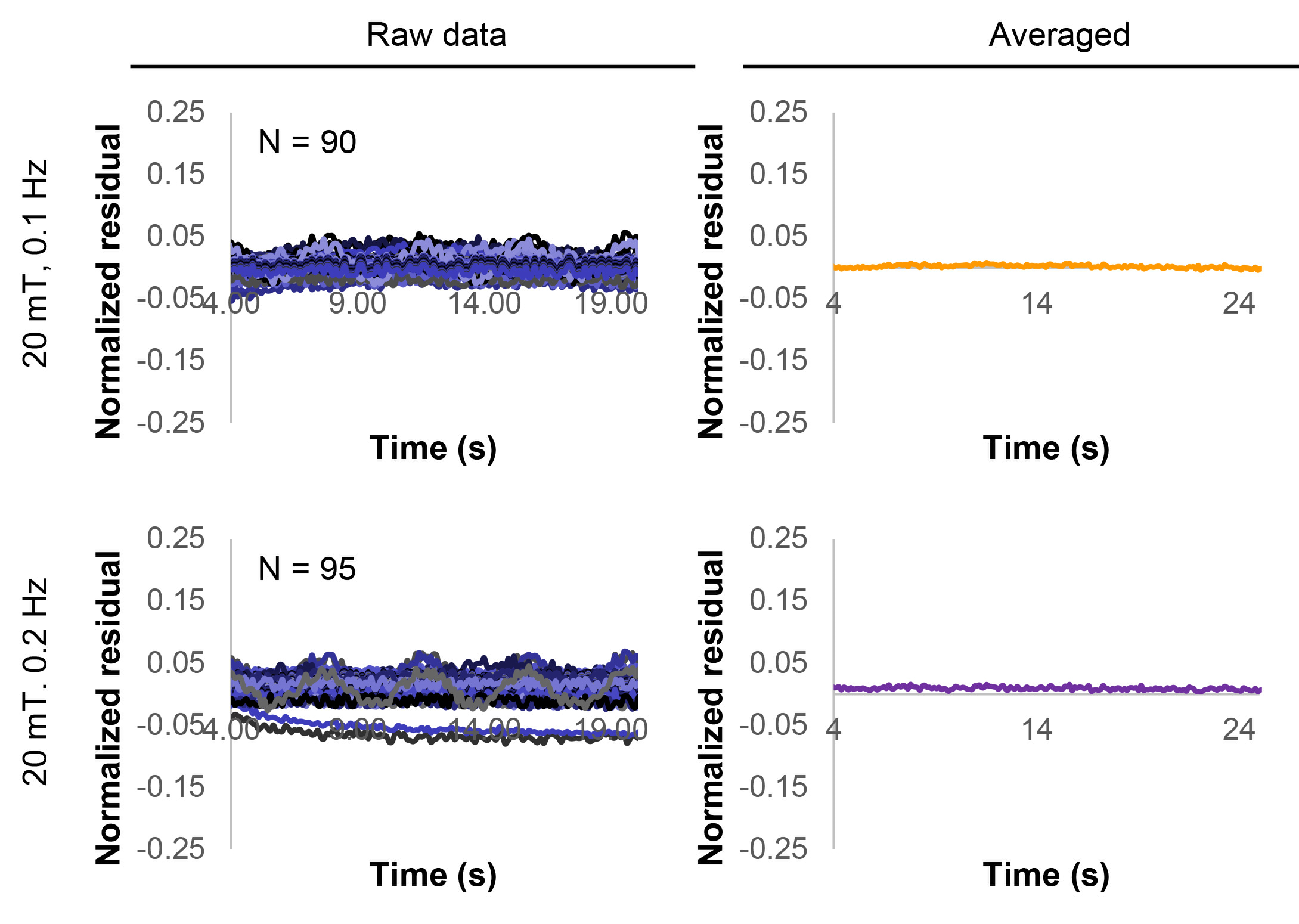


Figure S16. Raw data of normalized residuals of autoflourescent intensity response and corresponding averages. Cells were irradiated by 20 mT modulated magnetic field (frequencies 0.1 Hz and 0.2 Hz).


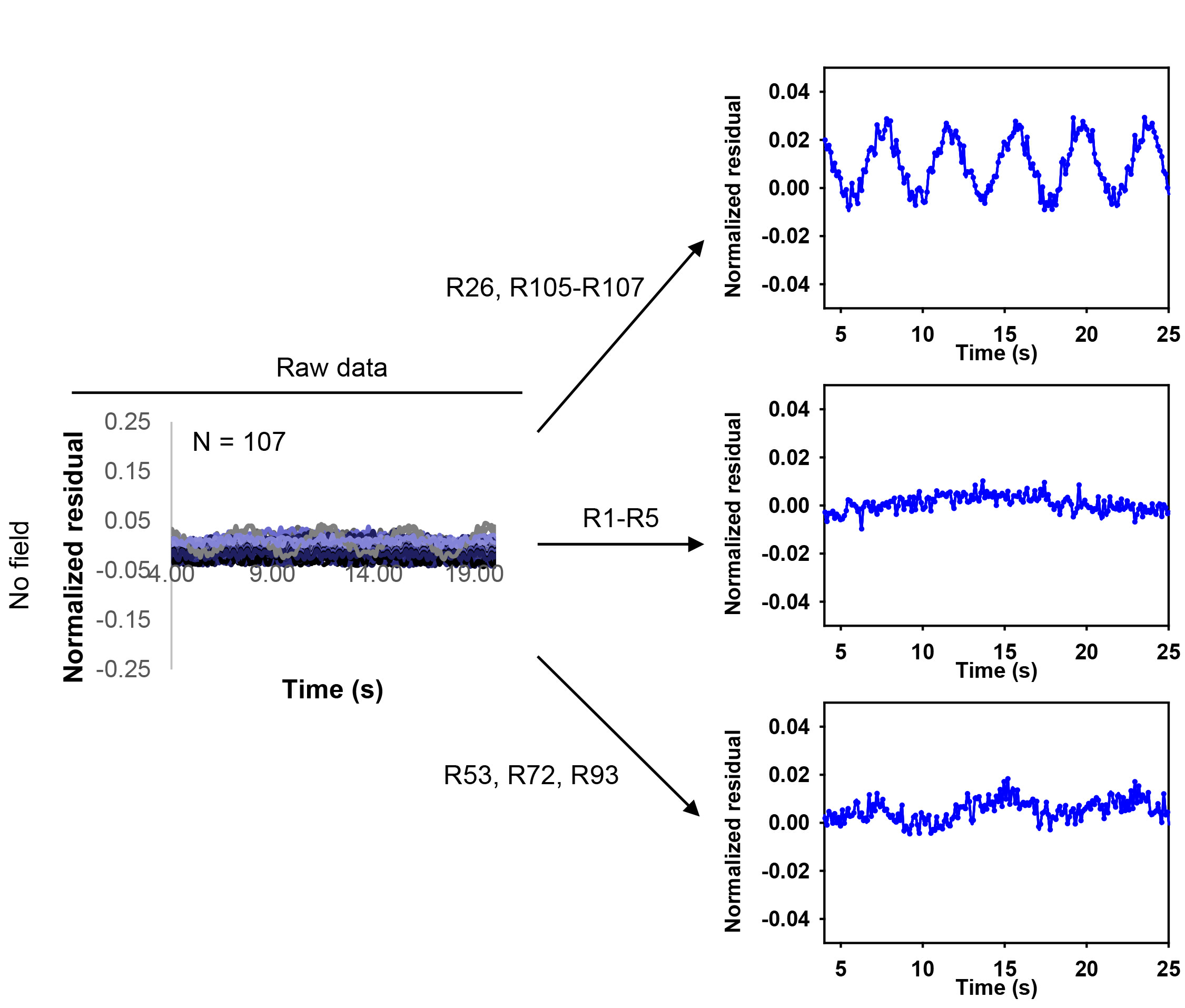


Figure S17. Normalized residuals of autoflourescent intensity of control cells (no field exposure). Selected cells represent different fluctuations of residuals without any magnetic field application.


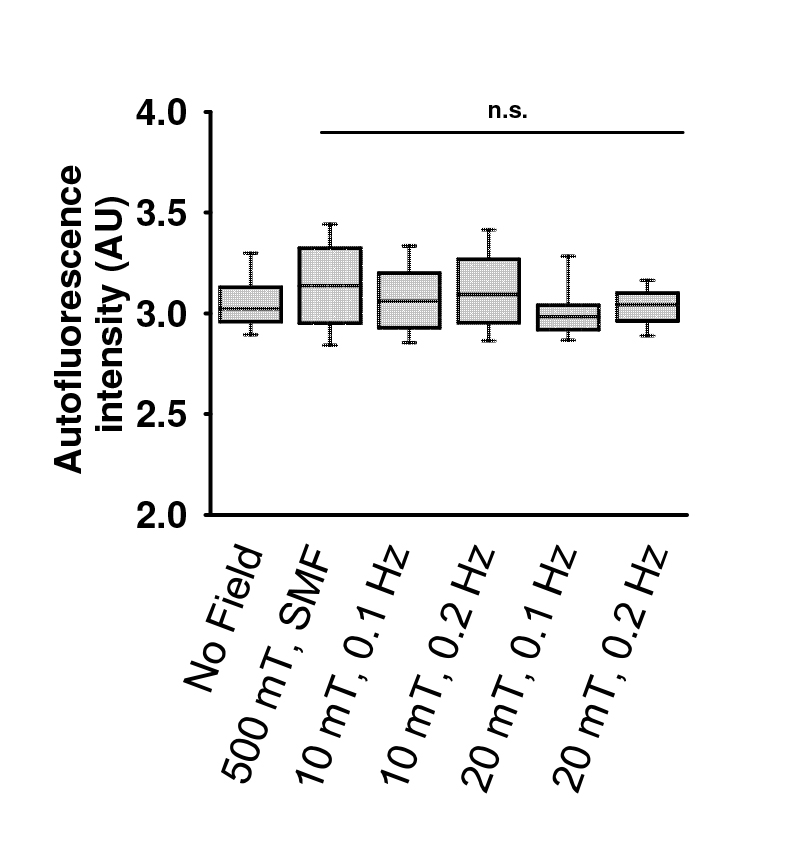


Figure S18. A box and whisker plot graphically represents the median, lower and upper quartiles, and lower and upper extremes of an autofluorescence intensity data. Cells were irradiated by 10 mT or 20 mT modulated magnetic field (frequencies 0.1 Hz and 0.2 Hz). 500 mT static magnetic field (SMF) was generated by bulk NdFeB magnet. N=90-107. Dunnett’s test was used to determine statistical significance. Differences were considered statistically significant at *P < 0.05.

Legends for Movies

Movie S1 (separate file). Video of a representative autofluorescence photobleaching of HeLa cells without magnetic field.

Movie S2 (separate file). Zoomed region of Movie S1, autofluorescence photobleaching of HeLa cells without magnetic field.

Movie S3 (separate file). Video showing particle tracking analysis upon autofluorescence photobleaching of HeLa cells without magnetic field.

Movie S4 (separate file). Video showing movable structures upon autofluorescence photobleaching of HeLa cells without magnetic field.

Movie S5 (separate file). Video showing non-movable and highly bleachable structures upon autofluorescence photobleaching of HeLa cells without magnetic field.

Movie S6 (separate file). Combined video showing particle tracking analysis upon autofluorescence photobleaching of HeLa cells without magnetic field.

**References**

1. L. Valle, F. E. Vieyra, C. D. Borsarelli, Hydrogen-bonding modulation of excited-state properties of flavins in a model of aqueous confined environment. *Photochem Photobiol Sci* **11**, 1051-1061 (2012).

2. M. A. Yakovleva*, et al.*, Fluorescence characteristics of lipofuscin fluorophores from human retinal pigment epithelium. *Photochem Photobiol Sci* **19**, 920-930 (2020).

3. A. C. Croce, G. Bottiroli, Autofluorescence spectroscopy and imaging: a tool for biomedical research and diagnosis. *Eur J Histochem* **58**, 2461 (2014).

4. J. M. Menter, Temperature dependence of collagen fluorescence. *Photochem Photobiol Sci* **5**, 403-410 (2006).

5. A. C. Croce, A. Ferrigno, G. Bottiroli, M. Vairetti, Autofluorescence-based optical biopsy: An effective diagnostic tool in hepatology. *Liver Int* **38**, 1160-1174 (2018).

6. J. Koziol, Studies on flavins in organic solvents I. Spectral characteristics of riboflavin, riboflavin tetrabutyrate and lumichrome. *Photochemistry and Photobiology* **5**, 41-54 (1966).

7. S. D. M. Islam, A. Penzkofer, P. Hegemann, Quantum yield of triplet formation of riboflavin in aqueous solution and of flavin mononucleotide bound to the LOV1 domain of Photl from Chlamydomonas reinhardtii. *Chemical Physics* **291**, 97-114 (2003).

8. W. Holzer, A. Penzkofer, M. Fuhrmann, P. Hegemann, Spectroscopic characterization of flavin mononucleotide bound to the LOV1 domain of Phot1 from Chlamydomonas reinhardtii. *Photochemistry and Photobiology* **75**, 479-487 (2002).

9. L. G. Whitby, A new method for preparing flavin-adenine dinucleotide. *Biochemical Journal* **54**, 437-442 (1953).

10. S. D. M. Islam, T. Susdorf, A. Penzkofer, P. Hegemann, Fluorescence quenching of flavin adenine dinucleotide in aqueous solution by pH dependent isomerisation and photo-induced electron transfer. *Chemical Physics* **295**, 137-149 (2003).

11. J. A. Lewis, J. C. Escalante-Semerena, The FAD-dependent tricarballylate dehydrogenase (TcuA) enzyme of Salmonella enterica converts tricarballylate into cis-aconitate. *Journal of Bacteriology* **188**, 5479-5486 (2006).

12. L. E. Lamb, J. D. Simon, A2E: A component of ocular lipofuscin. *Photochemistry and Photobiology* **79**, 127-136 (2004).

13. https://patents.google.com/patent/US20180221455A1/en?oq=15886640.

14. R. F. Chen, Measurements of Absolute Values in Biochemical Fluorescence Spectroscopy. *Journal of Research of the National Bureau of Standards Section C-Engineering and Instrumentation* **A 76**, 593-606 (1972).

15. H. Mach, C. R. Middaugh, R. V. Lewis, Statistical determination of the average values of the extinction coefficients of tryptophan and tyrosine in native proteins. *Anal Biochem* **200**, 74-80 (1992).

16. J. R. Lakowicz (2006) *Principles of fluorescence spectroscopy* (Springer, New York, NY).

17. M. C. Mota, P. Carvalho, J. Ramalho, E. Leite, Spectrophotometric analysis of sodium fluorescein aqueous solutions. Determination of molar absorption coefficient. *Int Ophthalmol* **15**, 321-326 (1991).

18. K. W. Wang, S. B. Hladky, Absence of effects of low-frequency, low-amplitude magnetic-fields on the properties of gramicidin-a channels. *Biophys. J.* **67**, 1473-1483 (1994).

19. N. C. Shaner*, et al.*, Improved monomeric red, orange and yellow fluorescent proteins derived from Discosoma sp red fluorescent protein. *Nat. Biotechnol.* **22**, 1567-1572 (2004).

20. https://www.ibsmagnet.com/knowledge/flussdichte.php.
